# Supplementary material for: Cloning of the wheat leaf rust resistance gene Lr47 introgressed from Aegilops speltoides
Source: Nat Commun. 2023 Sep 28;14:6072. doi: 10.1038/s41467-023-41833-2 (PMC10539295; doi:10.1038/s41467-023-41833-2)
Supplement: Supplementary file 1 — Supplementary Information [file 41467_2023_41833_MOESM1_ESM.pdf]

Cloning of the wheat leaf rust resistance gene *Lr47* introgressed from *Aegilops speltoides*

Li *et al.*

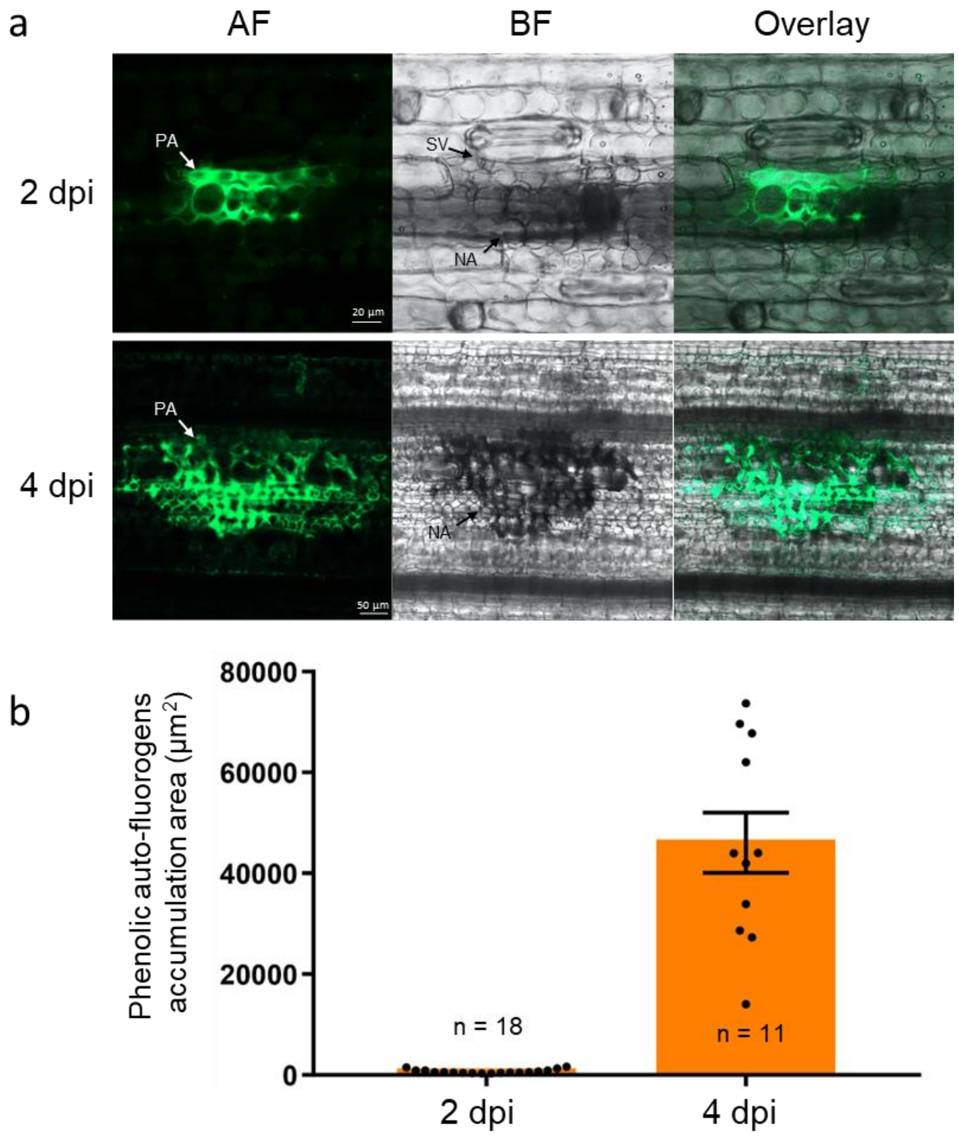

Supplementary Fig. 1. Histological observation of wheat leaves infected with *Pt* race THDB in Kern *Lr47*. a Infection sites were observed in leaf segments collected at 2 and 4 days post-inoculation (dpi). The same infection site was observed under both epi-fluorescence and bright field. Accumulation of phenolic auto-fluorogens was detected at 2 dpi and showed a significant increase at 4 dpi, indicating the activation of cell death or hypersensitive response by *Lr47* upon leaf rust infection. **b** Proportion of phenolic auto-fluorogens accumulation at each infection site was measured by microscopic software. Data were collected from at least ten independent infection sites (n=18 and n=11). Black dots represent single data points. Error bars are standard errors of the means. PA, phenolic auto-fluorogens (white arrows); SV, substomatal vesicle; NA, necrosis area; AF, auto-fluorescence; BF, bright field. Source data are provided as a Source Data file.

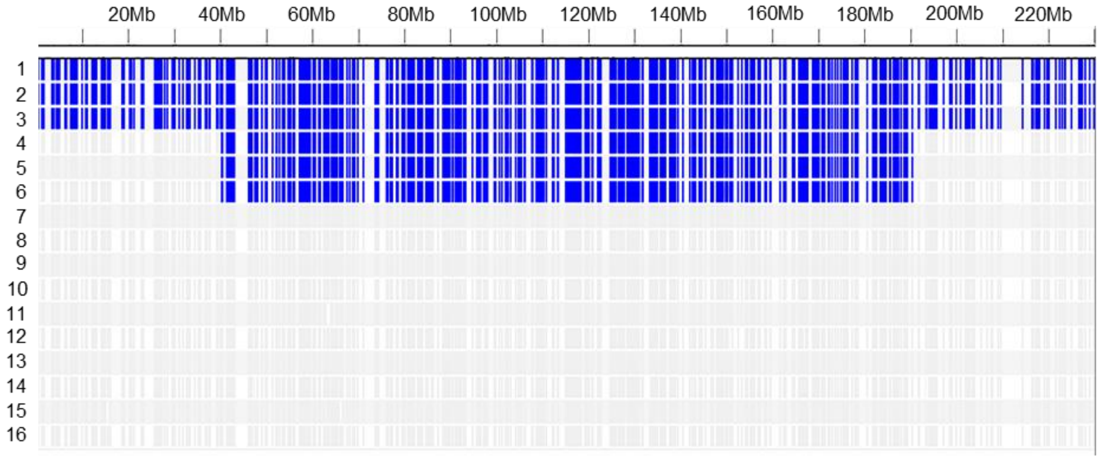

Supplementary Fig. 2. Distribution of 3,523 putative *Ae. speltoides*-specific SNPs on chromosome 7AS. The introgressed *Ae. speltoides* segment in Kern *Lr47*, Yecora Rojo *Lr47*, and UC1041 *Lr47* was approximately 150 Mb long, extending from 40 Mb to 190 Mb. 1-3) *Ae. speltoides* accessions AE915, AE1590, and PI 554292; 4-6) *Lr47* introgression lines Kern *Lr47*, Yecora Rojo *Lr47*, and UC1041 *Lr47*; 7-16) Sequenced *T. aestivum* accessions Norin61, CDC Stanley, Mace, Julius, Arina *LrFor*, Jagger, LongReach Lancer, CDC LandMark, SY Mattis, and Chinese Spring (Supplementary Data 2). Integrative Genomics Viewer (IGV) software version 2.8.9 [1] was used to visualize the distribution of these SNPs. Vertical lines in blue indicate *Ae. speltoides*-specific SNPs, while lines in light gray represent normal wheat SNPs. Coordinates are based on CS RefSeq v1.1.

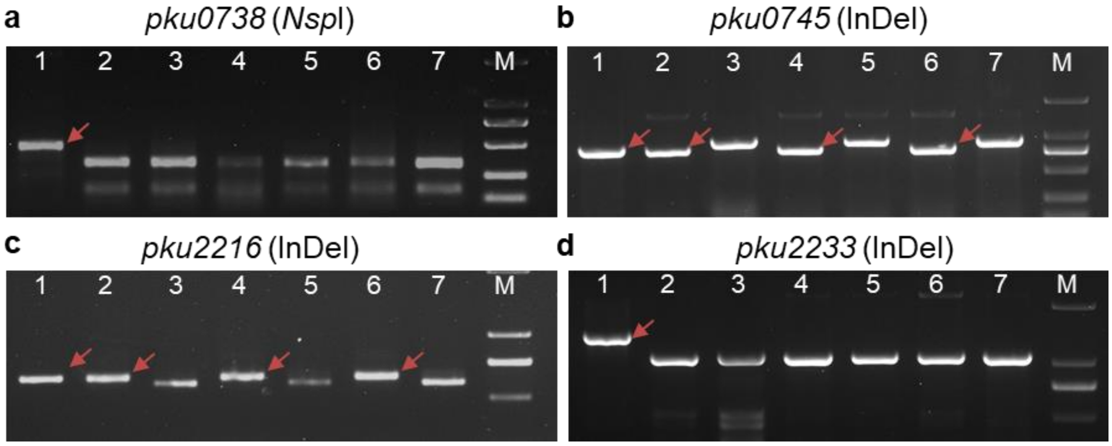

Supplementary Fig. 3. The introgressed *Ae. speltoides* segment is characterized by PCR markers. a CAPS marker *pku0738* digested with *Nsp*I (39.2 Mb); **b** InDel marker *pku0745* (40.2 Mb); **c** InDel marker *pku2216* (190.5 Mb); **d** InDel marker *pku2233* (192.7 Mb). 1, PI 554292 (*A. speltoides* control); 2, 4, 6) the *Lr47* NILs Kern *Lr47*, UC1041 *Lr47*, and RSI5 *Lr47*; 3, 5, 7) the recurrent parental lines Kern, UC1041, and RSI5. Red arrows represent *A. speltoides* bands. M, DNA ladder. Coordinates are based on CS RefSeq v1.1. Source data are provided as a Source Data file.

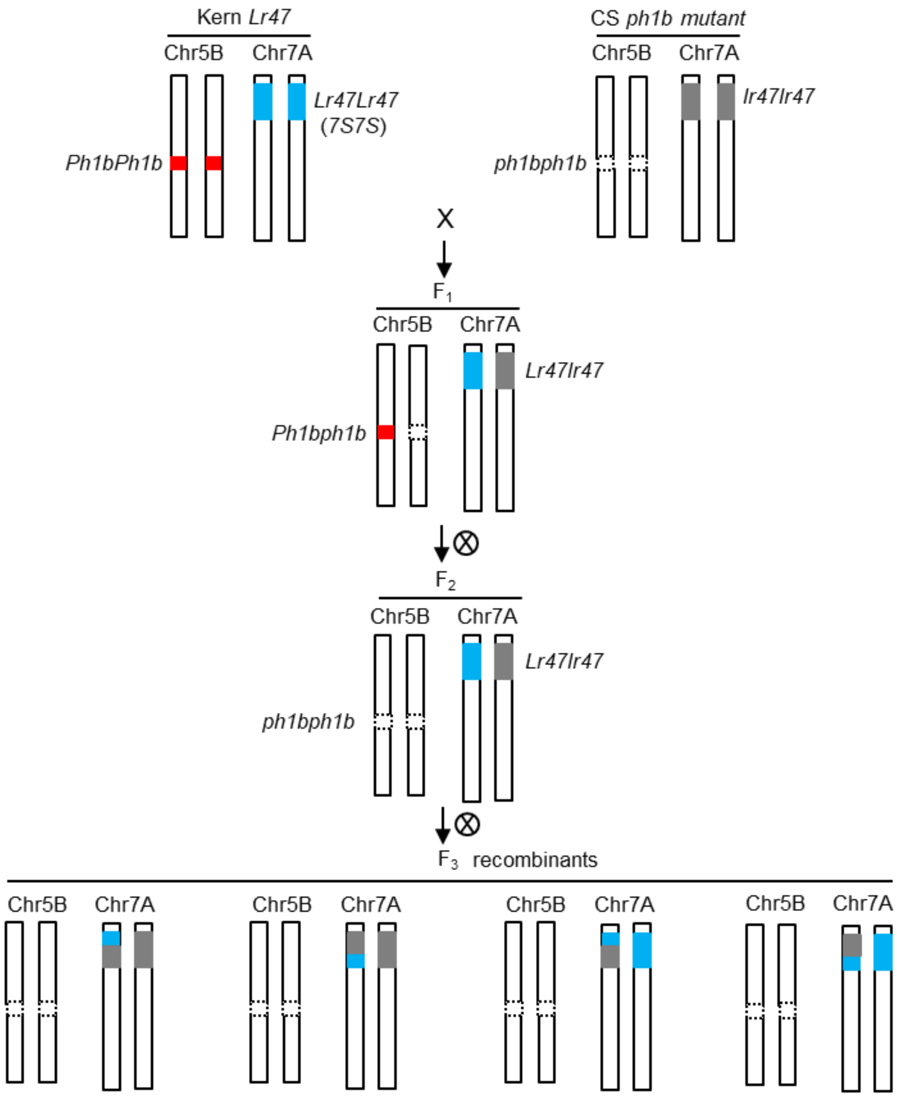

Supplementary Fig. 4. Procedure to reduce the length of the introgressed *Ae. speltoides* segment 7S#1S using the *ph1b* mutation. Blue rectangles indicate *Ae. speltoides* chromatin, and gray rectangles represent *T. aestivum* chromatin. Red boxes indicate the *Ph1b* region, and dotted boxes indicate the *ph1b* mutation (deletion).

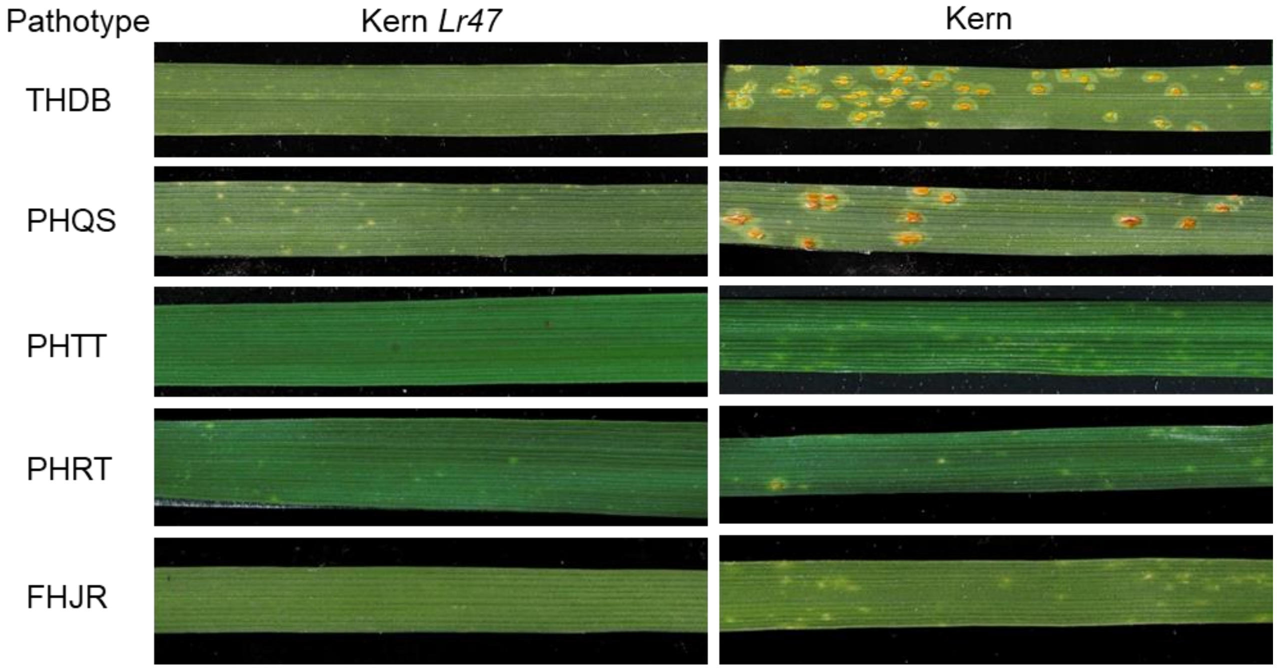

Supplementary Fig. 5. Seedling infection types of Kern *Lr47* and its recurrent parent Kern in response to *Pt* races THDB, PHQS, PHTT, PHRT, and FHJR. Plants were grown in a growth chamber at 24 °C during the day and 22 °C at night with a 16 h light /8 h dark photoperiod.

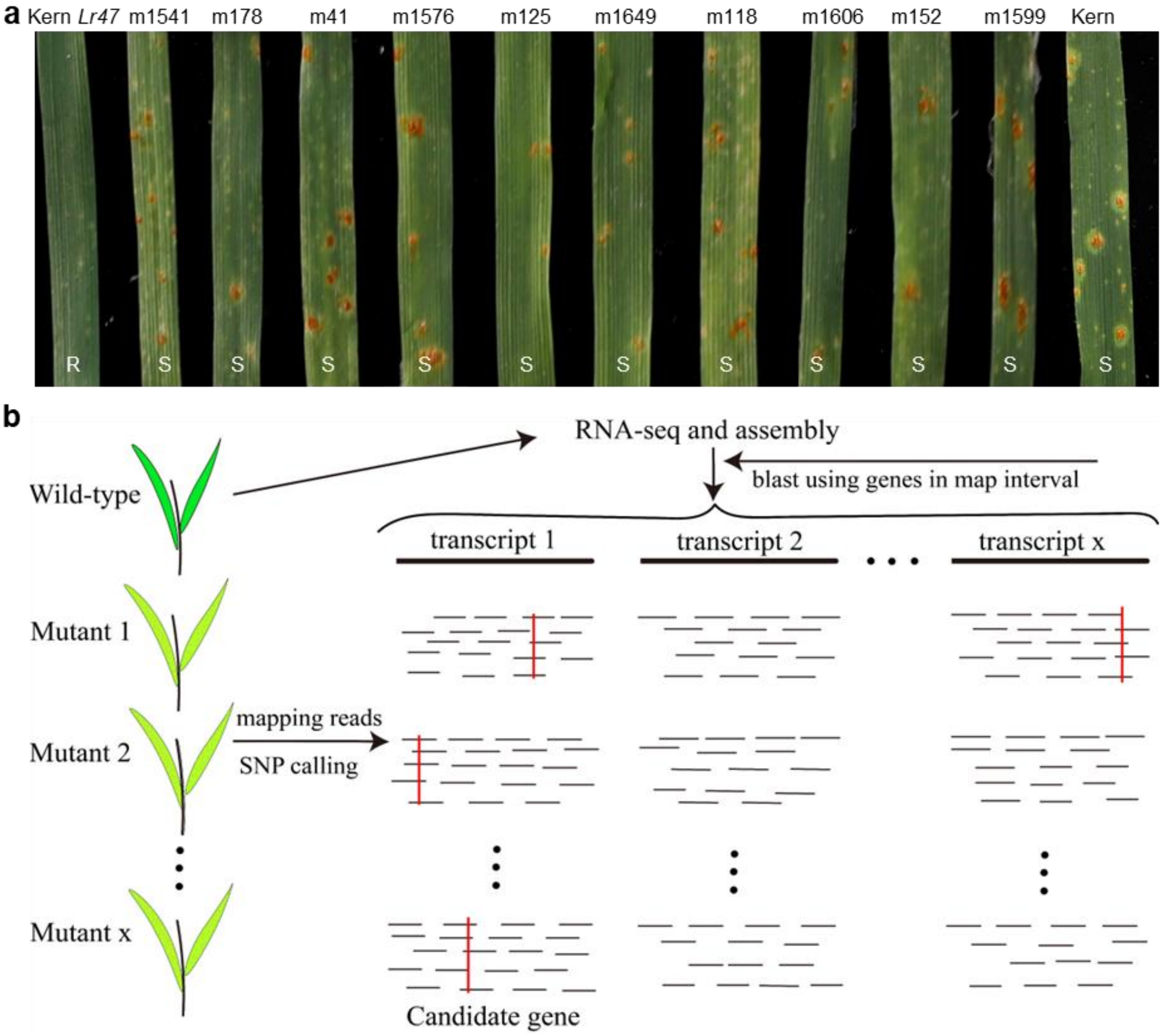

Supplementary Fig. 6. Identification of the *Lr47* candidate gene by modified MutRNASeq. a Infection types for Kern *Lr47* (positive control), ten independent EMS mutants, and Kern (negative control) inoculated with *Pt* race PHQS. R, resistant; S, susceptible. **b** Schematic representation of the modified MutRNASeq method. RNA-seq reads from the resistant parent (wild type) are *de novo* assembled. A search of the transcriptome database of the resistant parent using the sequences of genes within the mapping interval in sequenced reference genomes yields transcripts with high similarity. Next, RNA-seq reads from susceptible EMS mutants are mapped to the obtained transcripts. Transcript 1 in this example, which has a preponderance of single-nucleotide variants (red lines) across the susceptible mutants, is considered a good candidate.

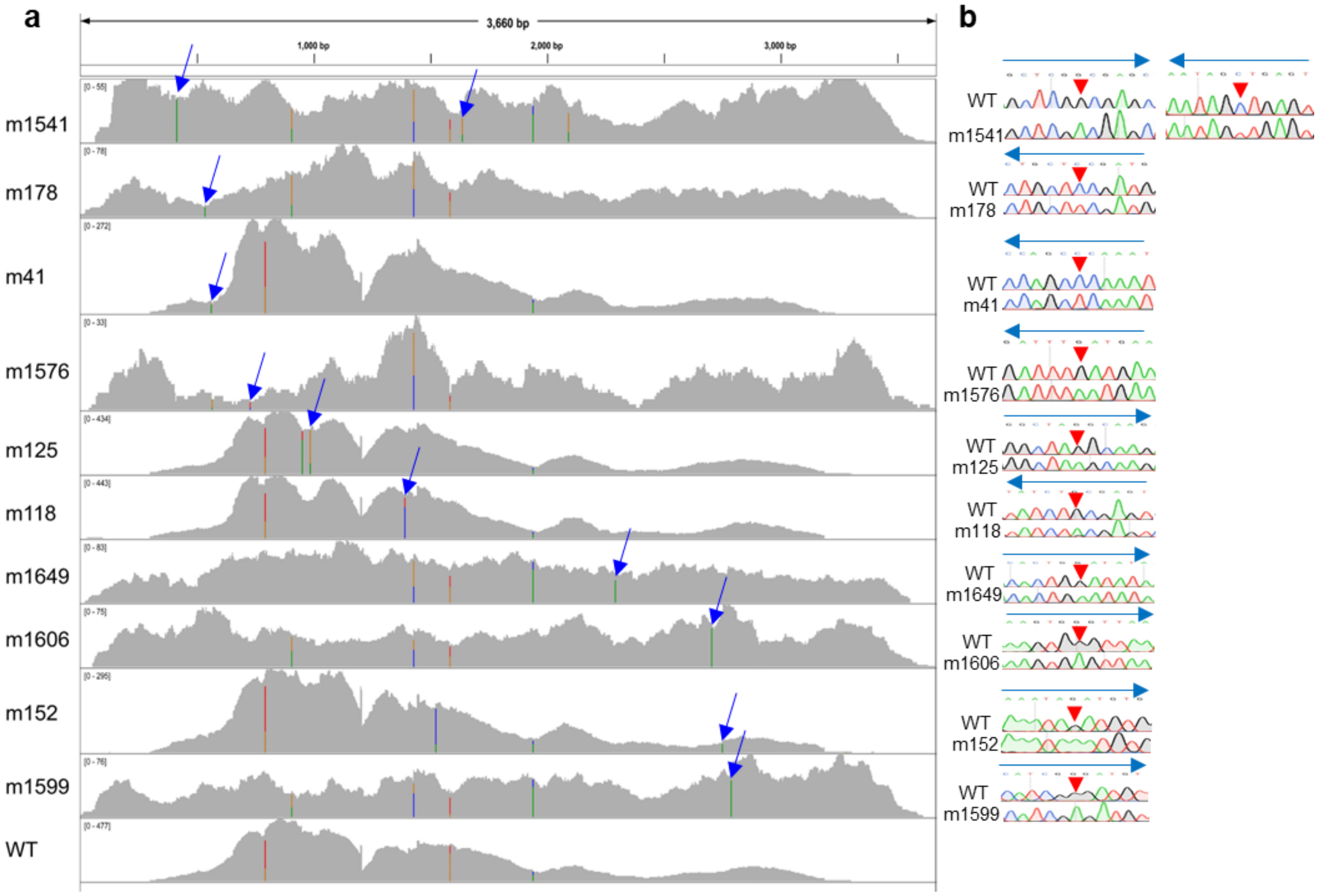

Supplementary Fig. 7. Integrative Genomics Viewer (IGV) snapshots for the *Lr47* candidate gene captured from MutRNASeq. a IGV snapshots showing the sequenced reads mapped to one transcript with EMS-type point mutations in all ten mutants. The EMS-type mutations are marked with blue arrows. m1541, m178, m41, m1576, m125, m118, m1649, m1606, m152, and m1599 are susceptible EMS mutants. WT, wild type (cv. Kern *Lr47*). **b** Sequencing chromatograms showing the nucleotide transitions in the susceptible mutants. The mutated nucleotides are highlighted by red arrowheads. Blue arrows, + strand/- strand.

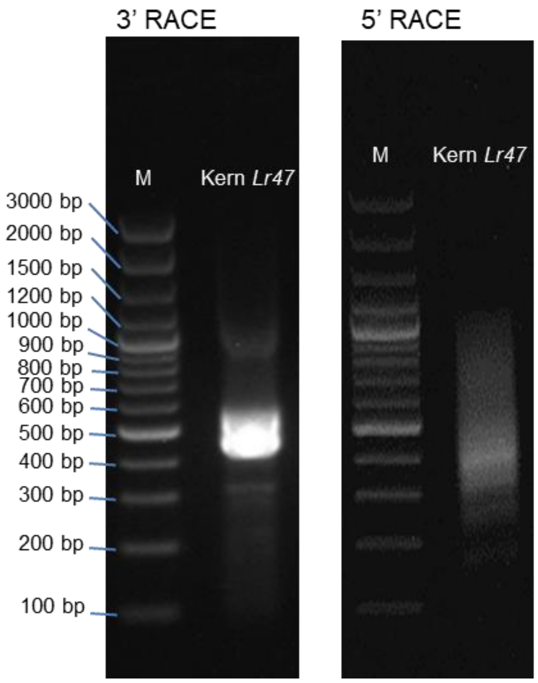

Supplementary Fig. 8. PCR products from RACE reactions. Primers developed at the 3′ and 5′ coding regions of the *Lr47* candidate gene were used as the gene-specific primers (Supplementary Data 3) for nested PCRs. The FirstChoice RLM-RACE Kit (ThermoFisher Scientific, MA; Cat. no. AM1700) was used to perform the RACE reactions.

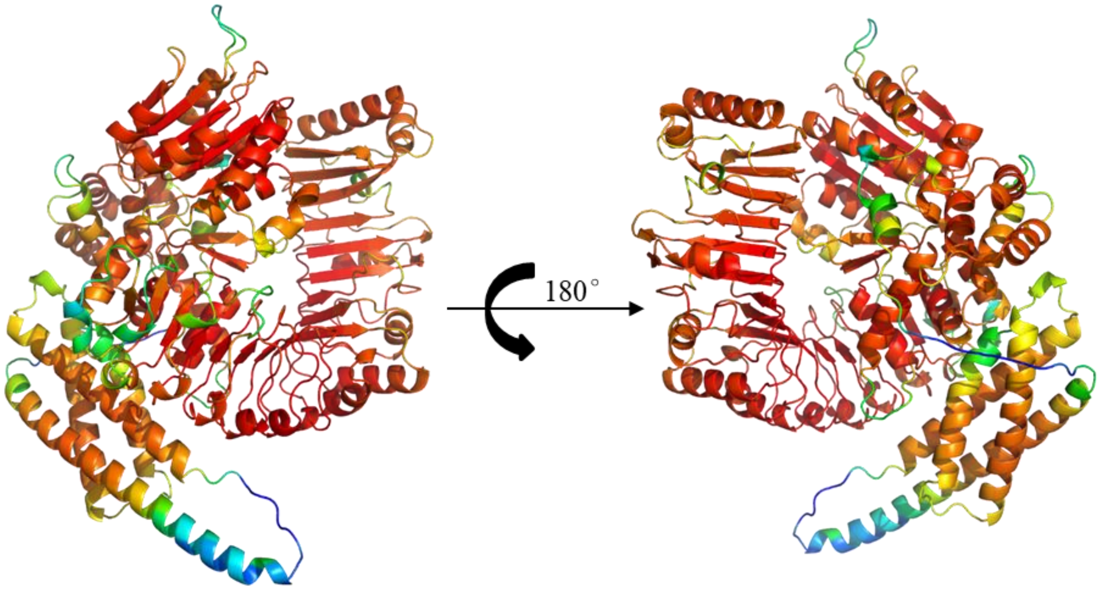

Supplementary Fig. 9. Structure of the Lr47 candidate predicted by AlphaFold v2.0.1 [2]. AlphaFold prediction of the full-length CNL2 protein yielded a structural model with the expected CC and NB domains as well as an LRR domain containing multiple repeat units that forms a typical α/β horseshoe fold.

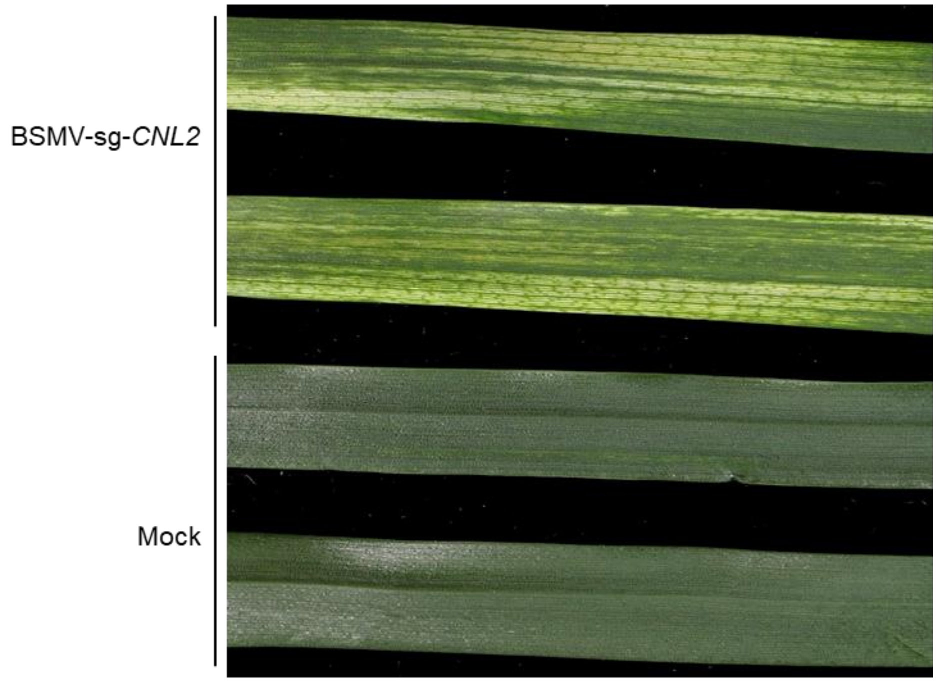

Supplementary Fig. 10. Representative phenotypes of the leaves from F_1 plants inoculated with the BSMV-sgRNA constructs targeting the candidate gene *CNL2*. Uninfected leaves (Mock) served as controls.

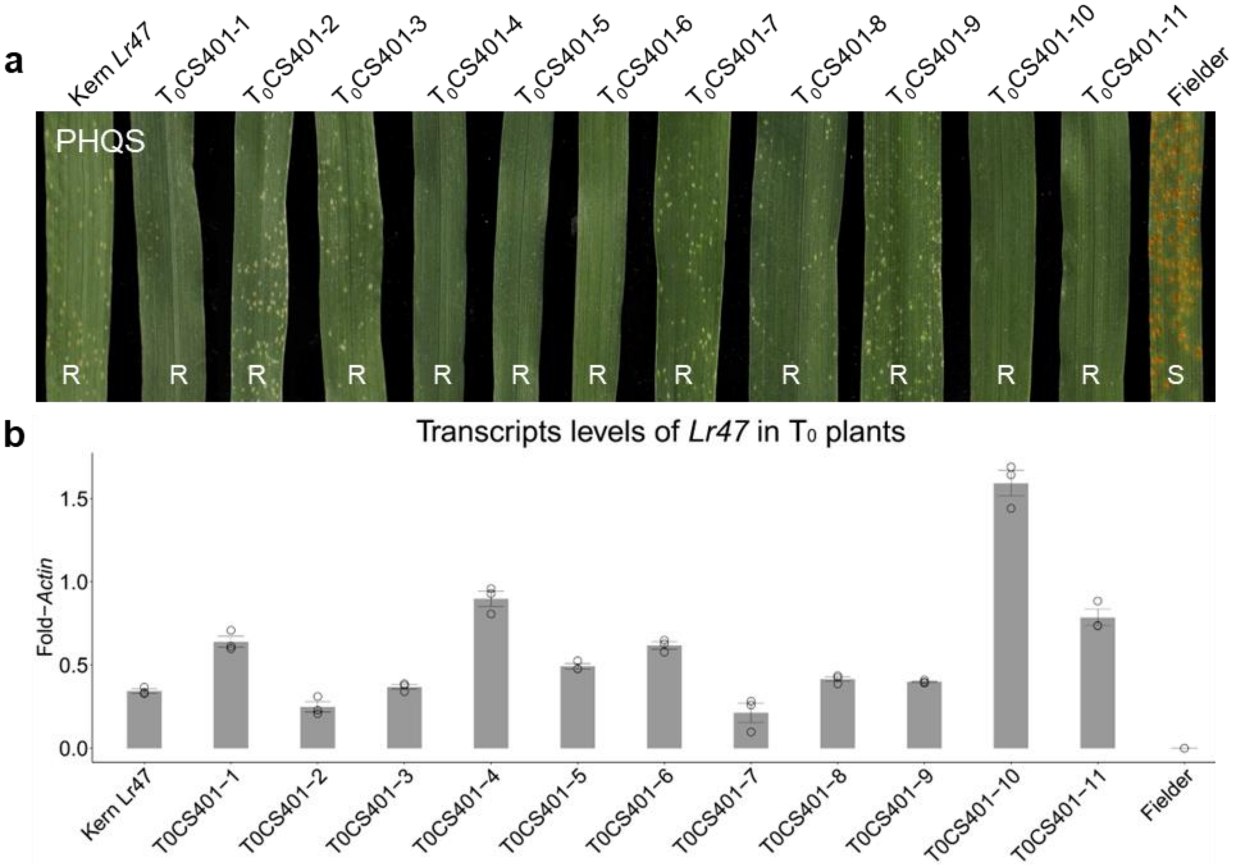

Supplementary Fig. 11. *Lr47* T_0 transgenic plants. a Reactions to *Pt* race PHQS in Kern *Lr47*, 11 randomly selected T_0 transgenic plants (T_0CS401-1 to T_0CS401-11), and Fielder. Plants were grown at 24 °C during the day and 22 °C at night. S, Susceptible; R, Resistant. **b** Transcript levels of *Lr47* in Kern *Lr47*, 11 T_0 transgenic plants, and Fielder. Transcript levels are expressed as fold-*ACTIN* using the $2^{-\Delta CT}$ method ($n = 3$; three technical replicates from a single plant). Gray open dots represent single data points. Error bars are standard errors of the mean. Source data are provided as a Source Data file.

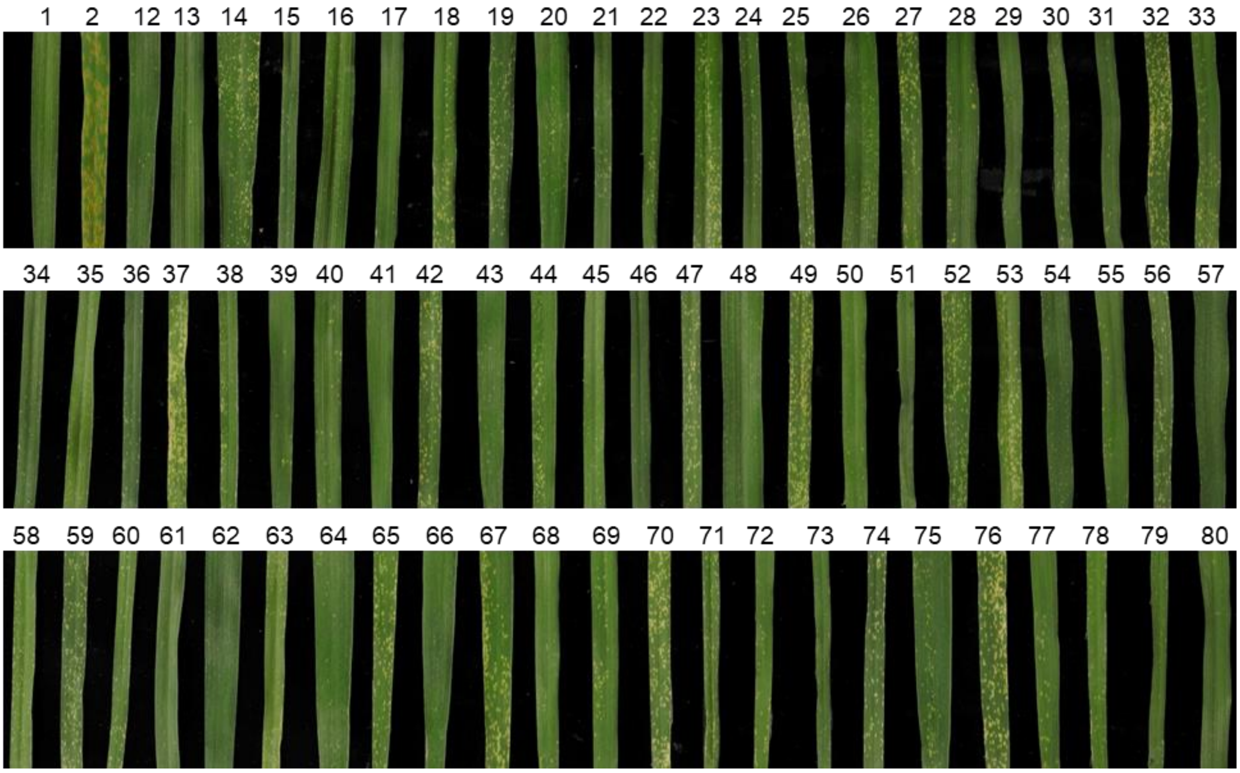

Supplementary Fig. 12. Reactions to *Pt* race PHQS of 1, Kern *Lr47*; 2, Fielder; and 12-80, another 69 T_0 transgenic plants (T_0CS401-12 to T_0CS401-80). Plants were grown in a growth chamber at 24 °C during the day and 22 °C at night with a 16 h light /8 h dark photoperiod.

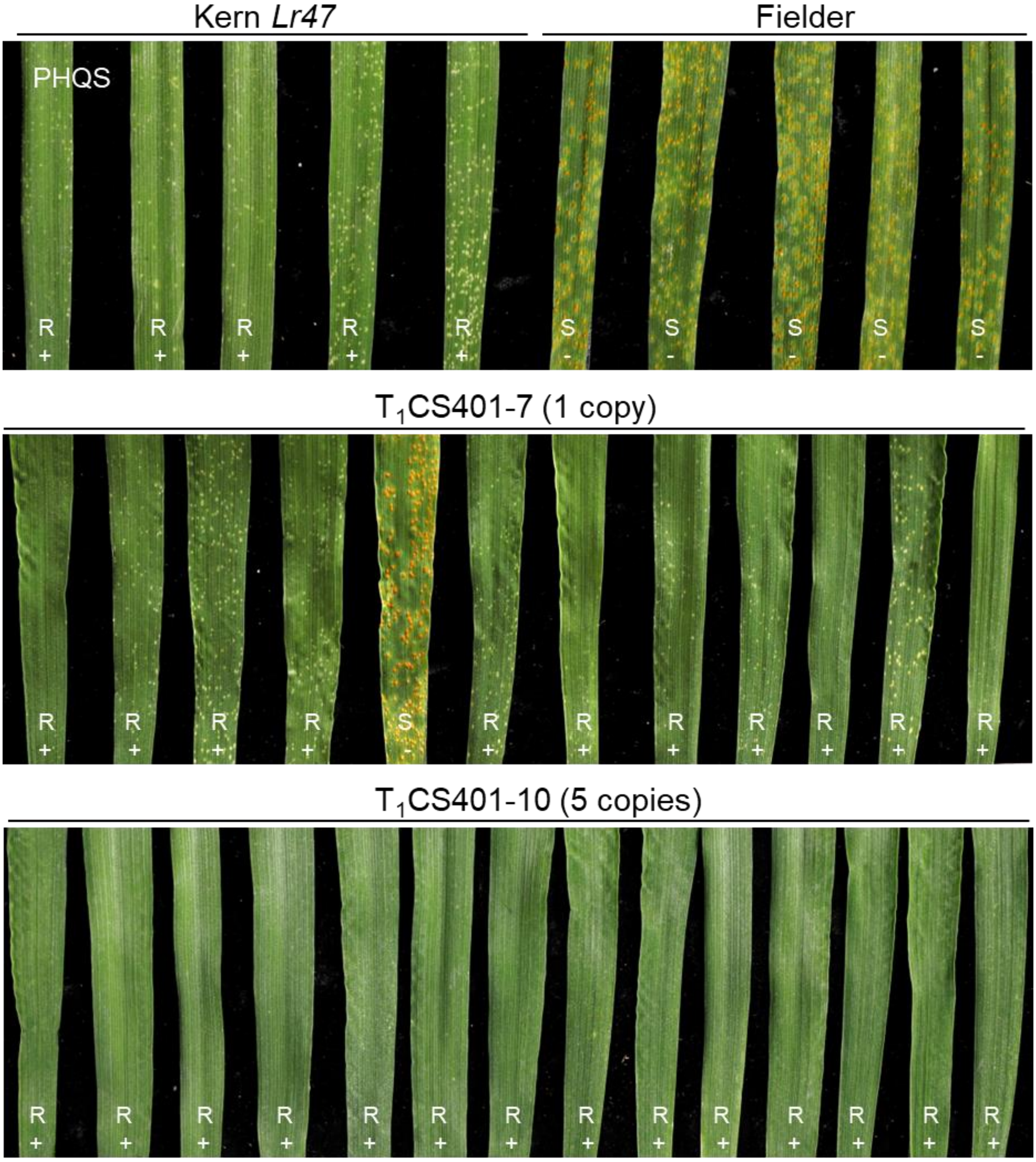

Supplementary Fig. 13. Reactions to *Pt* race PHQS in transgenic families T_1CS401-7 and T_1CS401-10. Family T_1CS401-7 was estimated to have a single copy of the transgene, while family T_1CS401-10 had five copies. Genotyping of the plants with marker *Lr47speF5R5* (Supplementary Data 3) revealed perfect co-segregation between the presence of transgene and the phenotypes. Kern *Lr47*, positive control; Fielder, untransformed control; S, susceptible; R, resistant; +, with transgene; -, without transgene.

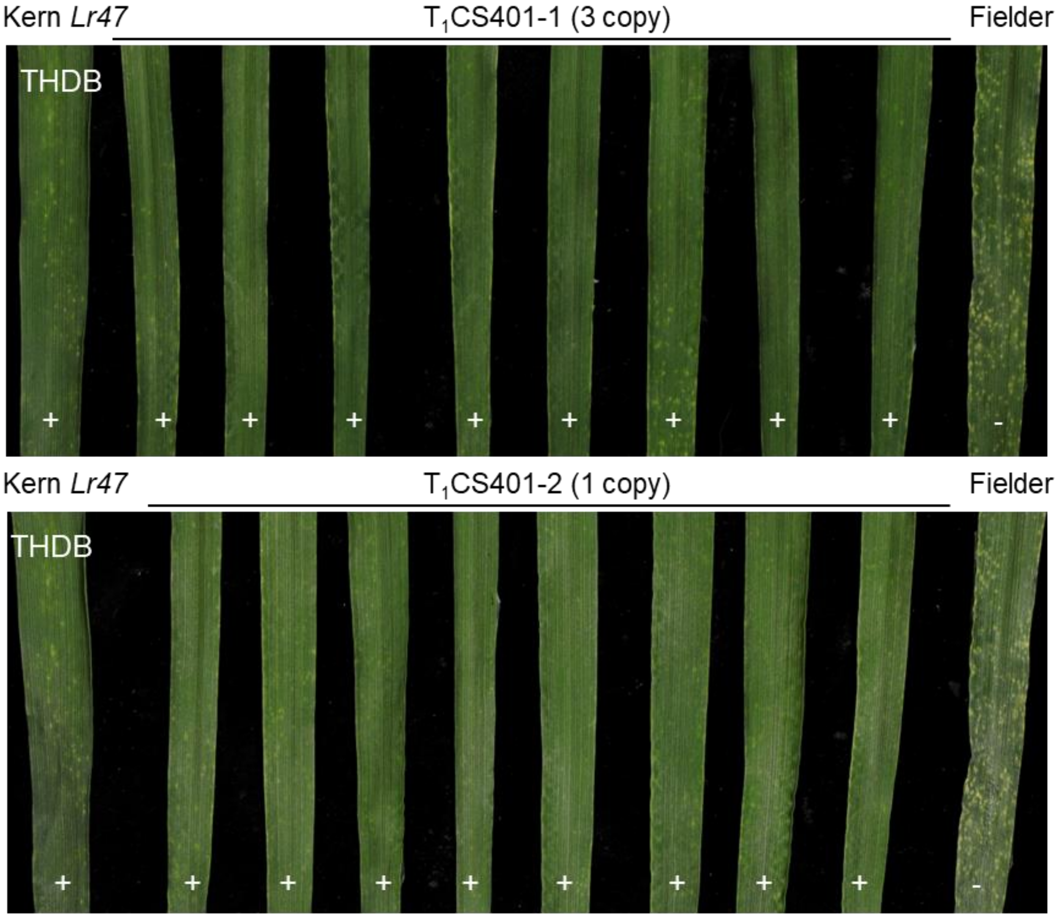

Supplementary Fig. 14. Inoculation of Kern *Lr47*, transgenic plants, and Fielder control with *Pt* race THDB. All T_1 plants in the T_1CS401-1 and T_1CS401-2 families were resistant to race THDB likely due to additional *Lr* gene(s) in the Fielder control that are resistant to this race. The "+" sign indicates the presence of the transgene and the "–" sign indicates its absence. Figure 4d in the main text presents the reactions of transgenic plants from the same families to *Pt* races FHJR, PHRT, PHTT, PHQS, FHJL, HCJR, THDB, and a mixture of naturally prevalent *Pt* races collected in 2021 from the field.

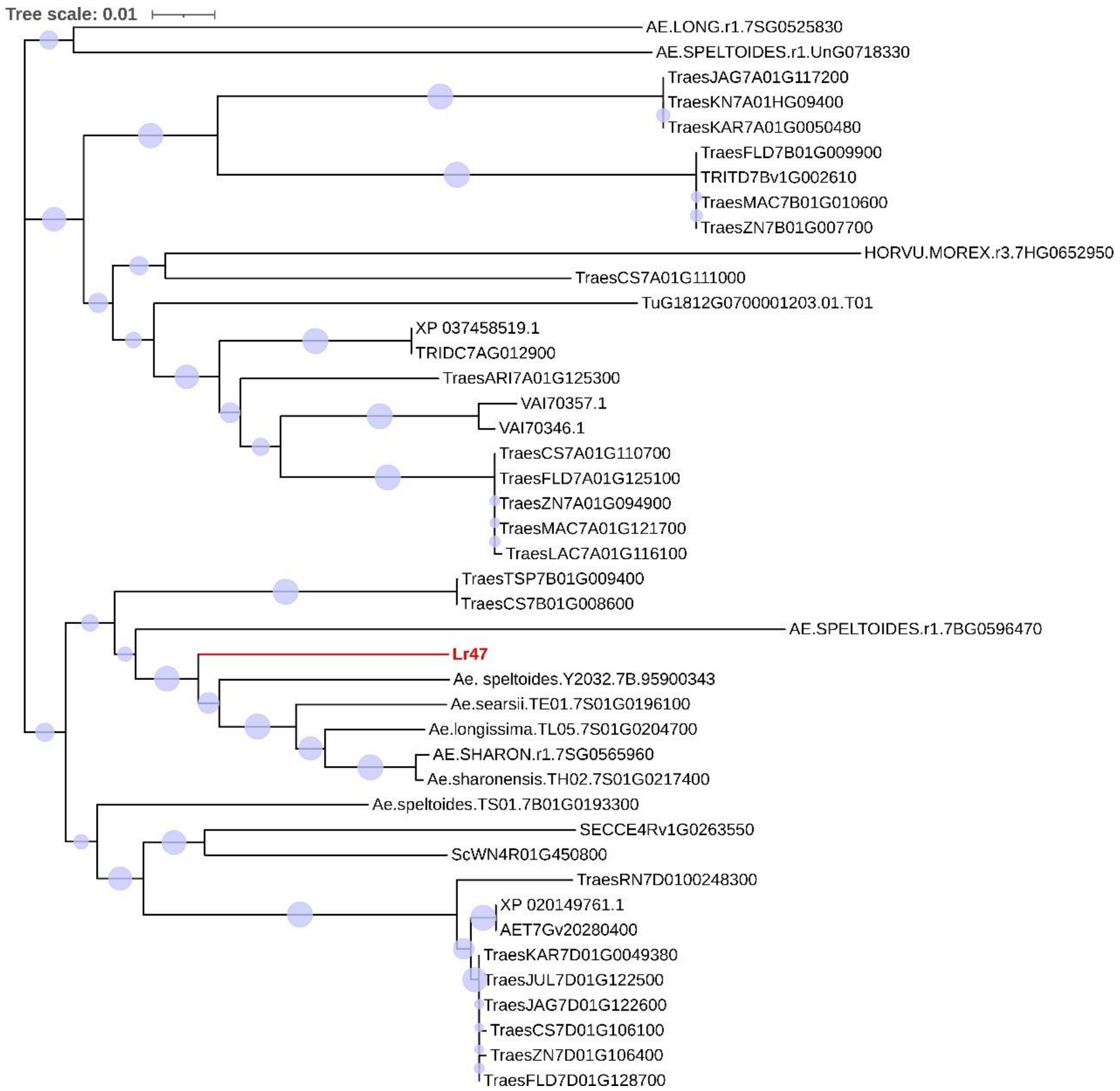

Supplementary Fig. 15. Phylogenetic tree of Lr47 related proteins. Neighbor-Joining tree of Lr47 protein and its homologs or similar proteins from *Triticum aestivum* (*Traes*), *T. urartu* (*Tu*), *T. dicoccoides* (*TRIDC*), *T. durum* (*TRITD*), *Secale cereale* (*Sc*), *Hordeum vulgare* (*HORVU*), and *Sitopsis* species (*Ae. speltoides*, *Ae. longissimi*, *Ae. sharonensis*, and *Ae. searsii*) calculated using MEGA 7. Other protein sequences were obtained from the National Center for Biotechnology Information (NCBI) website. Sequences were aligned using the muscle function implemented in MEGA 7, and phylogenetic trees were then generated using the pair-wise deletion method (bootstrap values based on 1000 iterations). Interactive Tree Of Life (iTOL) version 5 was used to visualize the tree (https://itol.embl.de/). (Note: For Ae. speltoides.Y2032.7B.95900343, frame shift mutation after amino acid 681 in the second exon. Insertion of 1-bp in this region restores similarity in the rest of the protein).

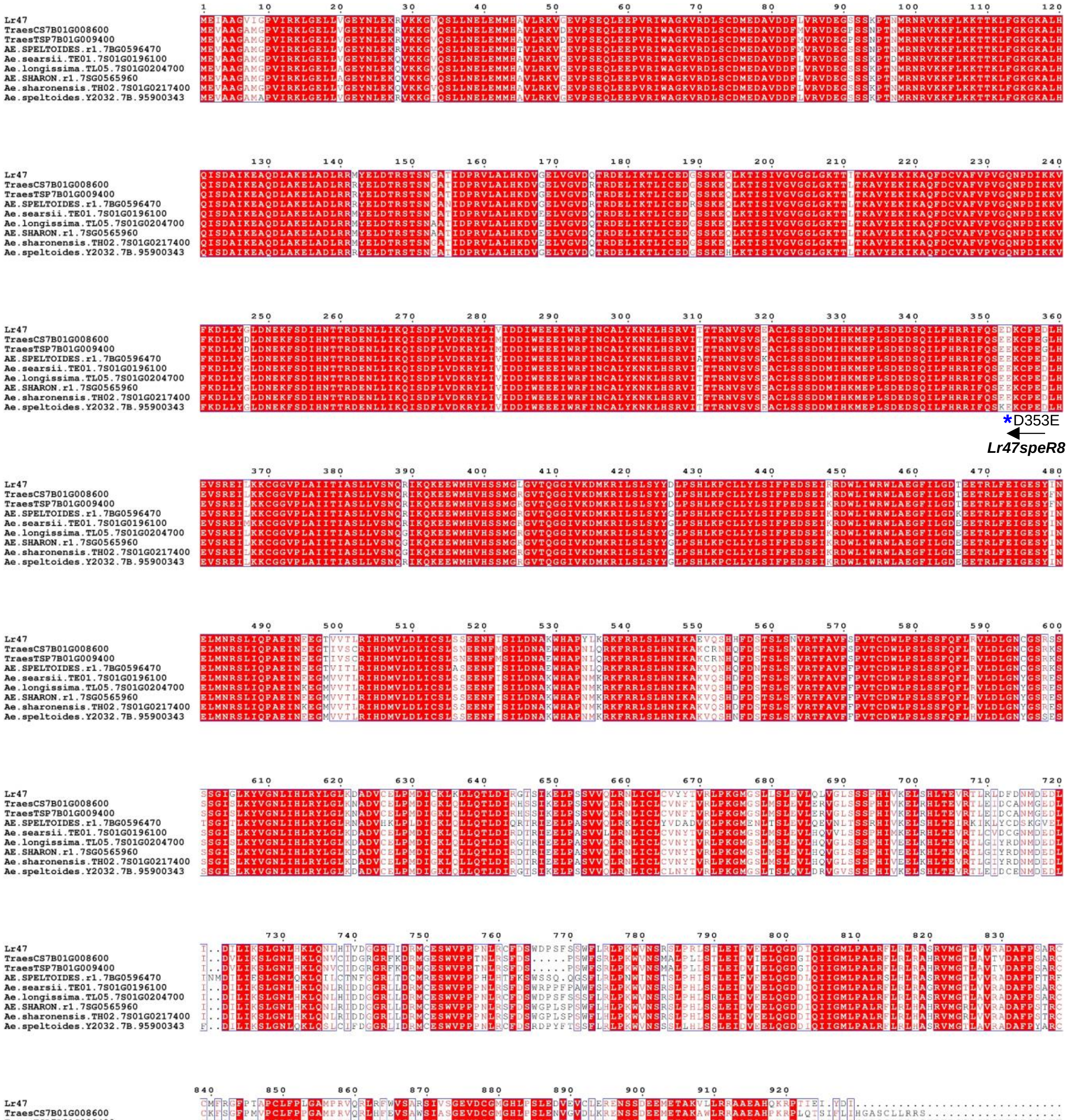

Supplementary Fig. 16. Sequence alignments of Lr47 protein and eight representative homologs. Highlighted in red are identical sequences among these proteins. Sequences were aligned using the muscle function implemented in MEGA 7. ESPript 3.0 was used to visualize the alignments (https://espript.ibcp.fr/ESPript/cgi-bin/ESPript.cgi). *, the DNA polymorphism (C1059G and the corresponding amino acid change D353E) was used to develop the diagnostic marker *Lr47mas* for *Lr47*. Black arrow, the position of the primer *Lr47speR8*.

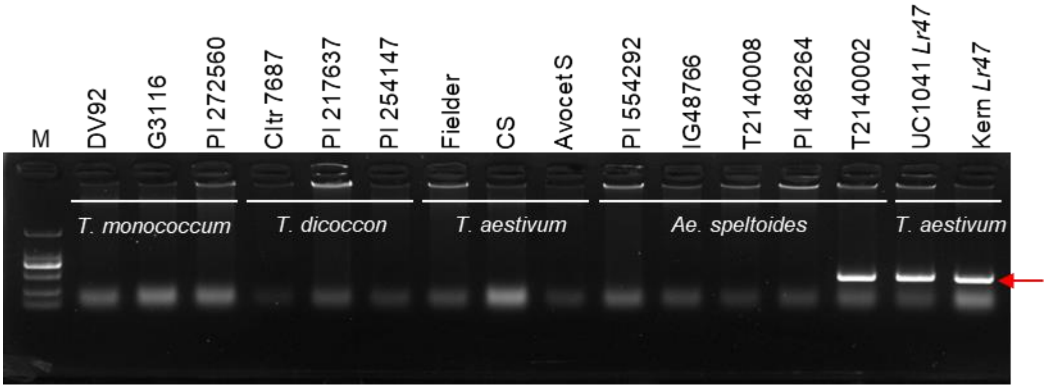

Supplementary Fig. 17. Amplification products of the *Lr47* diagnostic marker *Lr47mas*. The 488-bp PCR products (red arrow) are present only in the *Lr47* introgression lines and the *Ae. speltoides* accession T2140002 which carries *Lr47* (Supplementary Table 4). No PCR product was found in any of the tested lines of *T. monococcum* (e.g., DV92, G3116 and PI 272560), *T. turgidum* (e.g., CItr 7687, PI 217637, and PI 254147), and *T. aestivum* (e.g., Fielder, CS and Avocet S). PI 554292, IG48766, T2140008, and PI 486264 are *Ae. speltoides* accessions lacking *Lr47*. Source data are provided as a Source Data file.

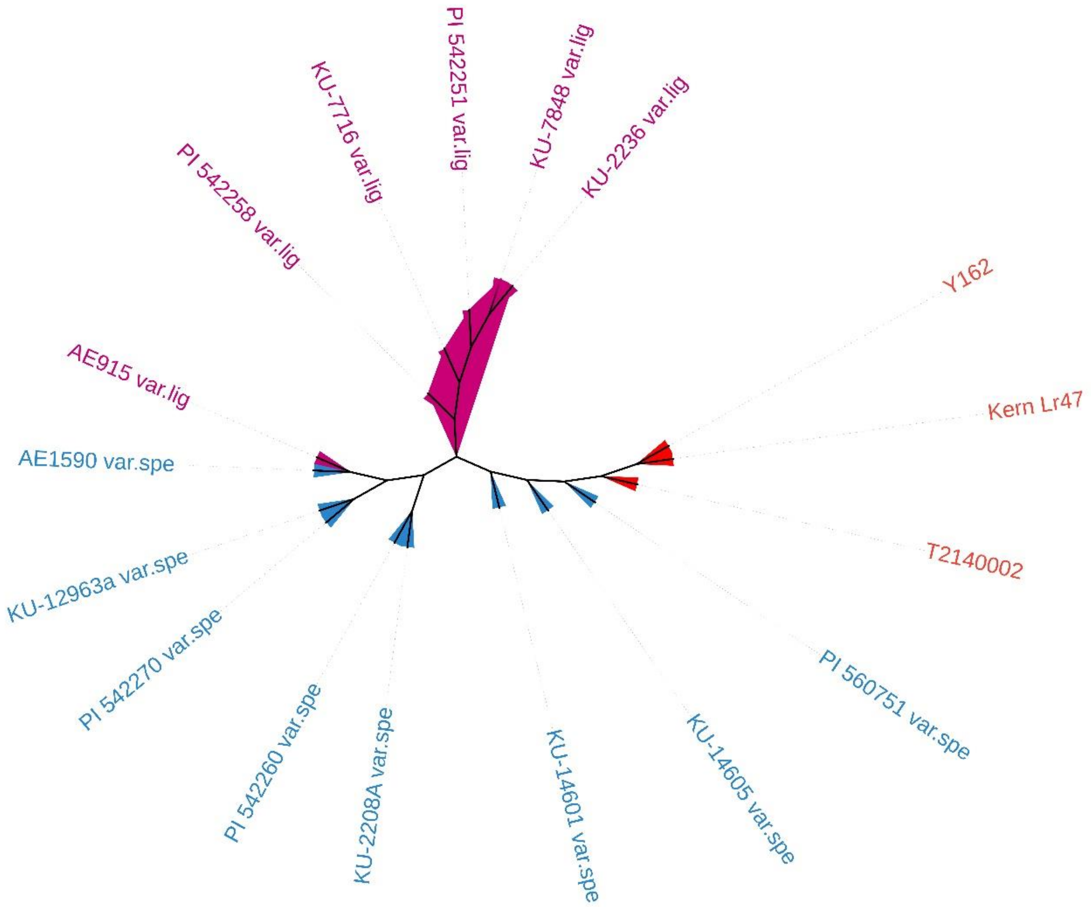

Supplementary Fig. 18. SNP-based phylogenetic analysis. Phylogenetic tree was constructed based on 199 polymorphisms (Supplementary Data 6) identified within the ~150 Mb introgressed segment 7S#1S from RNA-seq. The evolutionary history was inferred using the Neighbor Joining (NJ) method. Interactive Tree Of Life (iTOL) version 5 was used to visualize the tree (https://itol.embl.de/). Kern *Lr47*, Y162 and T2140002 are highlighted in orange; var. spe, *Aegilops speltoides* var. *speltoides* (highlighted in blue); var. lig, *Aegilops speltoides* var. *ligustica* (highlighted in purple).

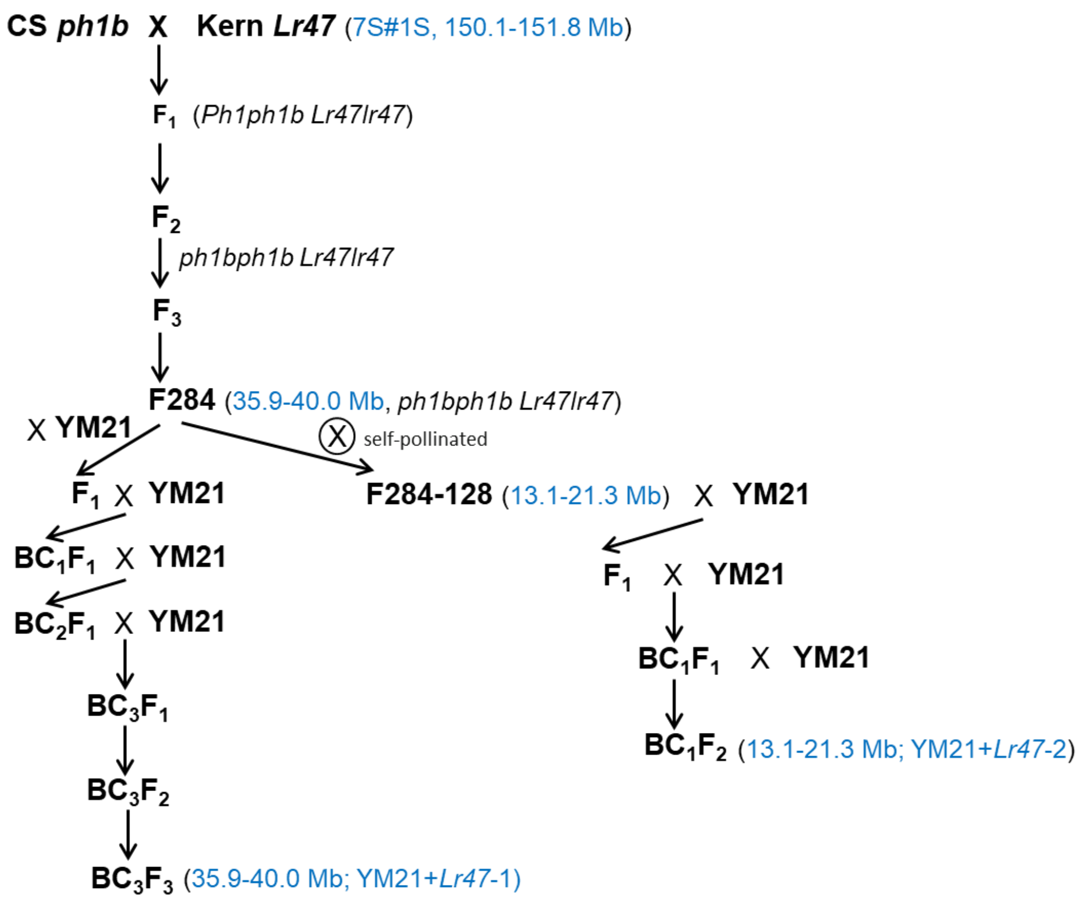

Supplementary Fig. 19. Procedure to reduce the size of the introgressed alien segment 7S#1S carrying *Lr47*. The 7A/7S-genome specific markers (Supplementary Data 3) distributed along the introgressed *Ae. speltoides* segment were used to monitor the length of alien chromatin. The length of alien chromatin was estimated based on CS RefSeq v1.1 and highlighted in blue.

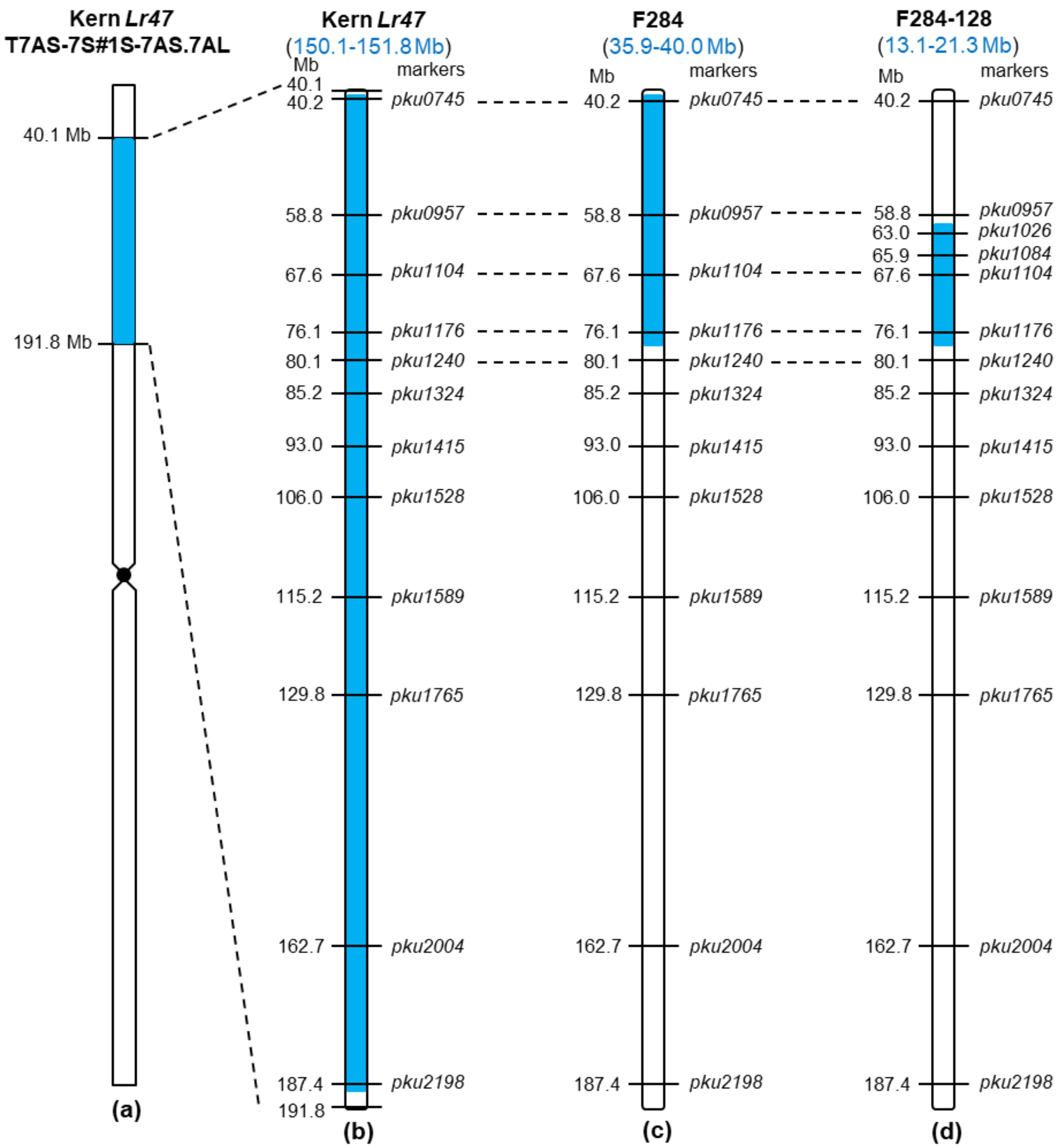

Supplementary Fig. 20. The genome specific markers used to analyze the length of the introgressed chromosomal fragment from *Ae. speltoides*. a Schematic representation of chromosome 7A in Kern *Lr47* carrying the introgressed *Ae. speltoides* segment 7S#1S (150.1-151.8 Mb, highlighted in blue). **b** The genome specific markers (Supplementary Data 3) distributed along the introgressed segment 7S#1S were used to estimate the length of alien chromatin. **c** The length of introgressed *Ae. speltoides* chromosome segment fragment in recombinant F284 (L2: 35.9-40.0 Mb; highlighted in blue). **d** The length of introgressed *Ae. speltoides* chromosome segment in recombinant F284-128 (L8: 13.1-21.3 Mb; highlighted in blue). Coordinates are based on CS RefSeq v1.1.

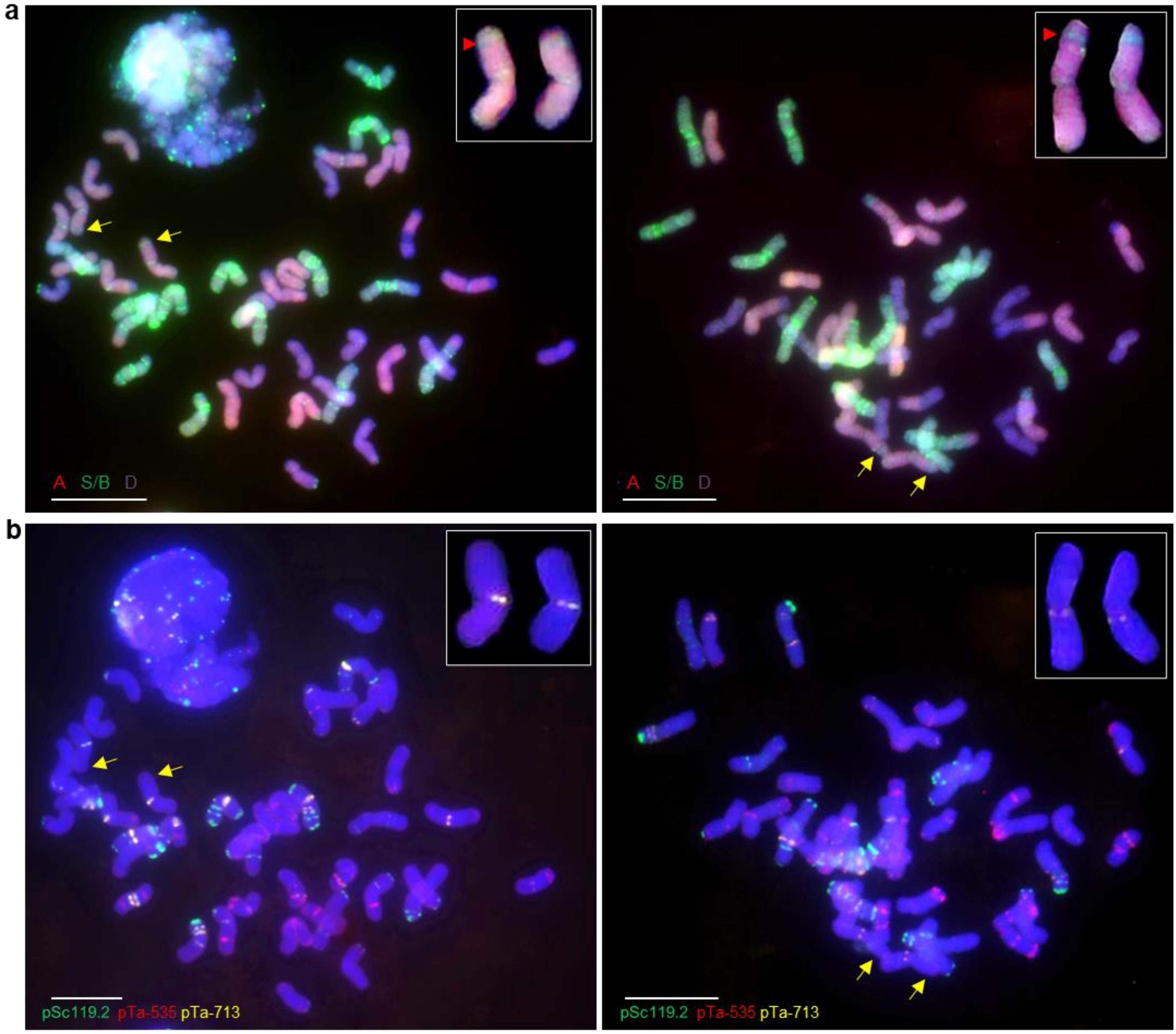

Supplementary Fig. 21. GISH and FISH images of the new introgression line YM21+*Lr47*-2 (L8, BC_1F_2 plants). **a** GISH pattern of mitotic chromosomes in wheat line YM21+*Lr47*-2. The magnified images show the *Ae. speltoides* chromosome segment introgressed into wheat chromosome 7A (*Ae. speltoides* chromatin is painted in green and marked with red arrowheads). Two mitotic metaphase cells of YM21+*Lr47*-2 were randomly observed. **b** FISH pattern of mitotic chromosomes in wheat line YM21+*Lr47*-2. Probes pSc119.2 (green), pTa535 (red), and pTa713 (yellow) were used in the current study. Yellow arrows indicate the wheat-*Ae. speltoides* translocated 7A chromosomes. Scale bar = 10 µm. All experiments were repeated three times independently with consistent results.

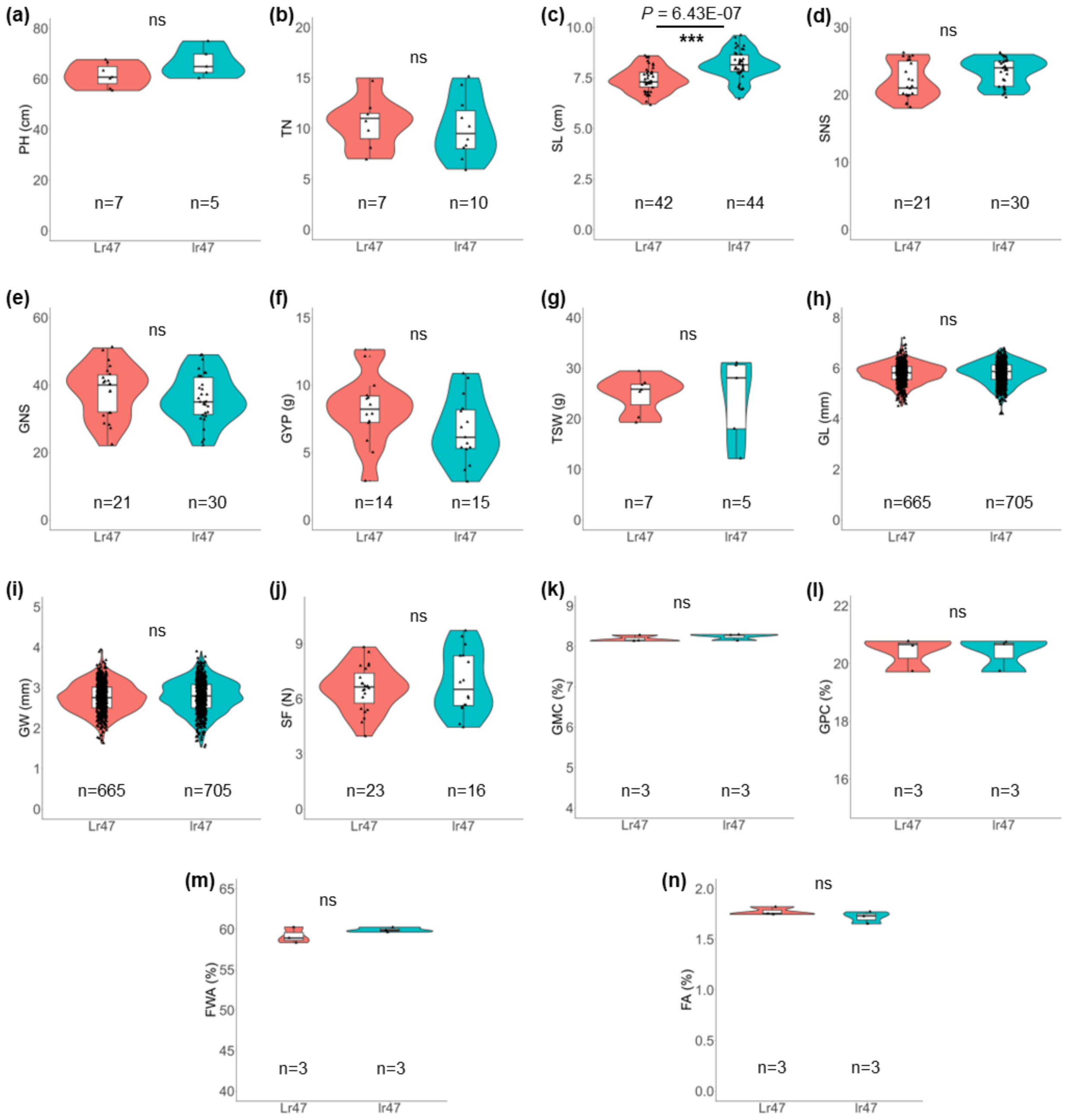

Supplementary Fig. 22. Statistical analysis of agronomic and quality traits in YM21+*Lr47*-1 and its sister control line. YM21+*Lr47*-1 and its sister control line were grown in a greenhouse (22-30 °C day and 16-24 °C night with a 16 h light/8 h dark photoperiod). **a** plant height (PH); **b** tillers number (TN); **c** spike length (SL); **d** spikelet number per spike (SNS); **e** grain number per spike (GNS); **f** grain yield per plant (GYP); **g** thousand-seed weight (TSW); **h** grain length (GL); **i** grain width (GW); **j** shearing force (SF); **k** grain moisture content (GMC); **l** grain protein content (GPC); **m** flour water absorption (FWA); and **n** flour ash (FA). The number of samples (n) are shown. In each box plot, the horizontal line shows the median (center, black line), box edges show the 25th/75th percentile, and whiskers are drawn to the minimum or maximum values within 1.5 times the interquartile range. The shape of the violin plot reflects the distribution of the variable. Black triangles represent single data points. The significance of the differences was estimated using two-sided unpaired *t*-test. ns = not significant ($P > 0.05$), ***, $P < 0.001$. Source data are provided as a Source Data file.

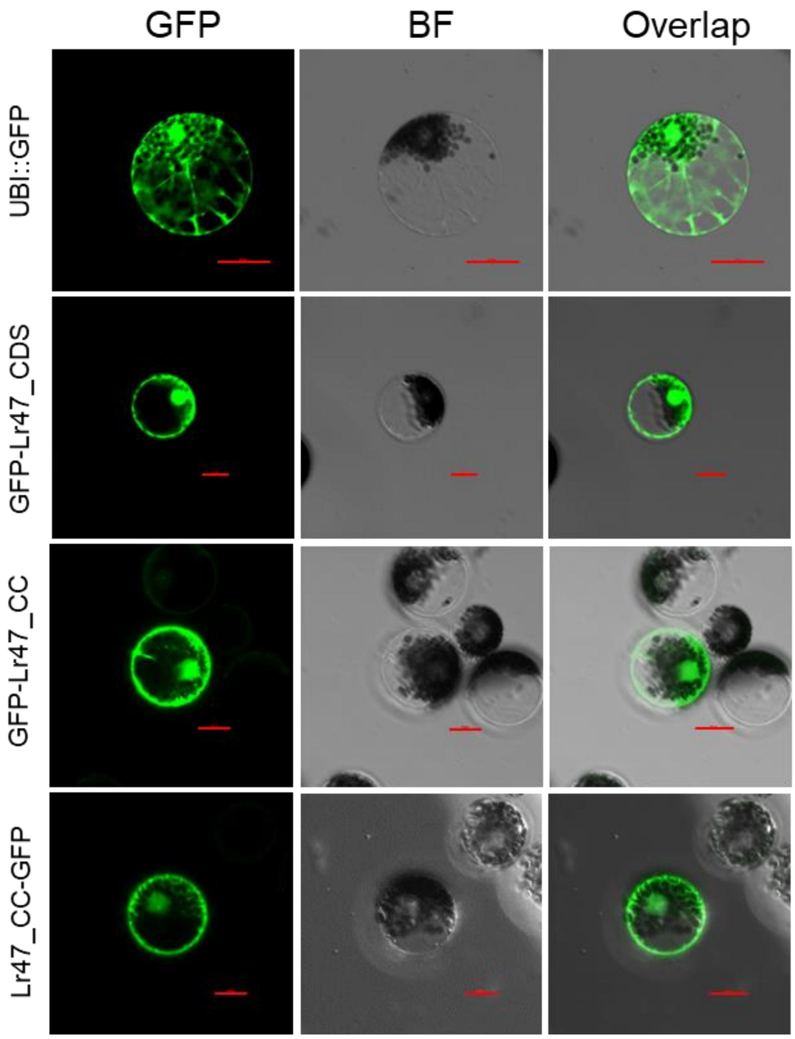

Supplementary Fig. 23. Subcellular localization of the Lr47 protein and its CC domain in wheat protoplasts. BF, bright field; GFP, green fluorescent protein. Scale bars (red lines) represent 10 µm. This experiment was repeated three times with consistent results.

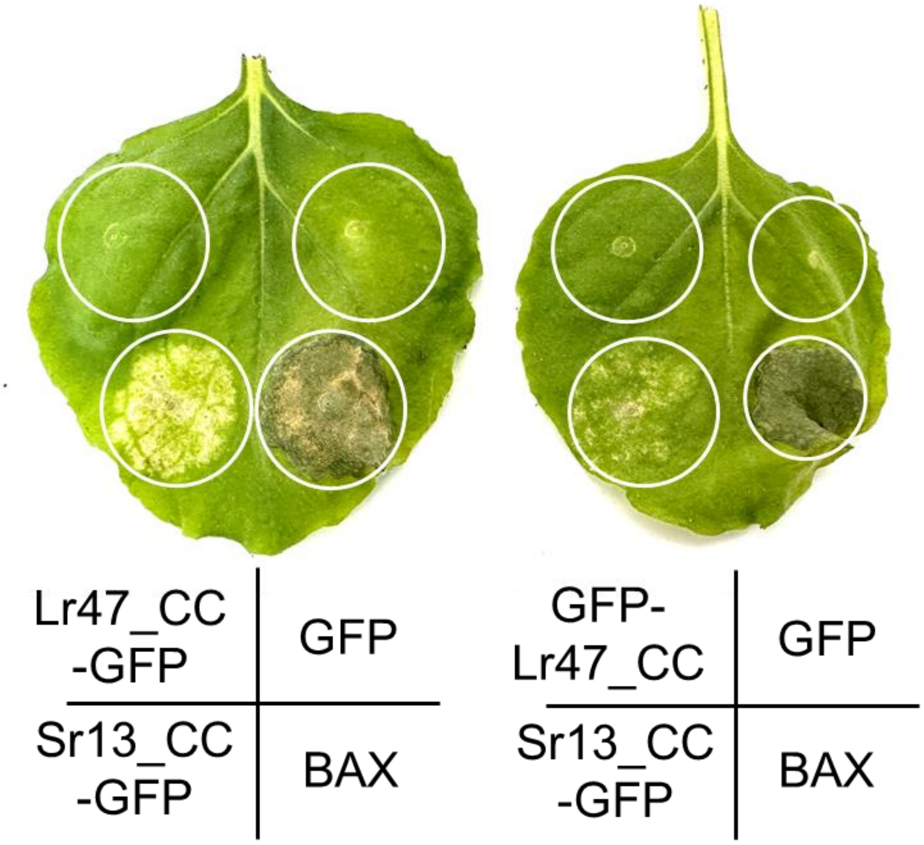

Supplementary Fig. 24. Macroscopic cell death in *N. benthamiana* leaves 48 h post infiltration (hpi) with the CC domain of Lr47. No cell death was observed in leaf regions transiently overexpressing the CC domain of Lr47. CC, coiled-coil; GFP was used as a negative control, while BAX and Sr13_CC were used as positive controls.

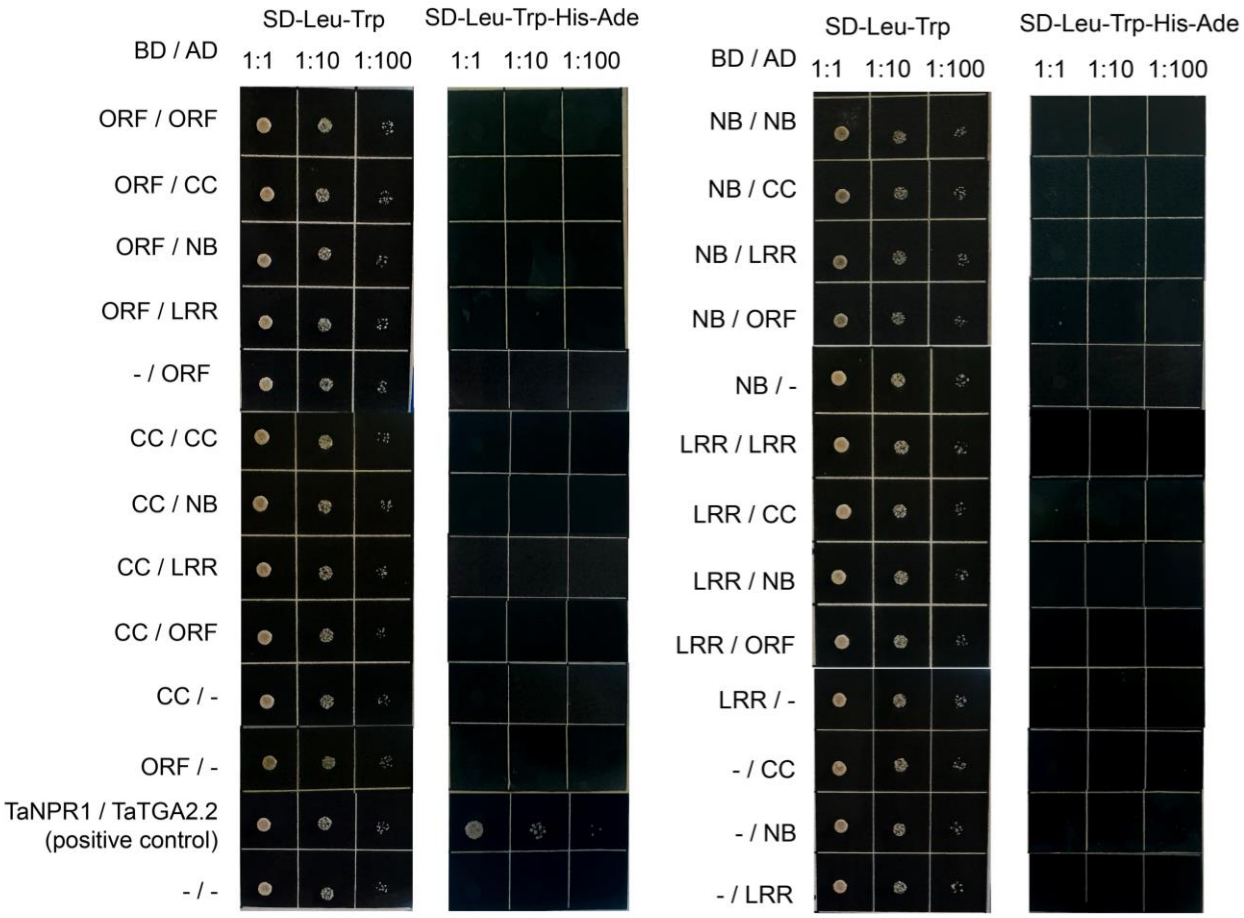

Supplementary Fig. 25. Self interactions among Lr47 and its conserved domains were investigated using yeast two-hybrid assay. Yeast transformants co-expressing different bait and prey recombinant constructs were tested on synthetic dropout medium lacking leucine and tryptophan (SD-Leu-Trp) for selection of transformed colonies and then leucine, tryptophan, histidine, and adenine (SD-Leu-Trp-His-Ade) to detect interactions. CC, coil-coil; NBS, nucleotide-binding site; LRR, leucine rich repeat; ORF, open reading frame (CDS); -, empty vector.

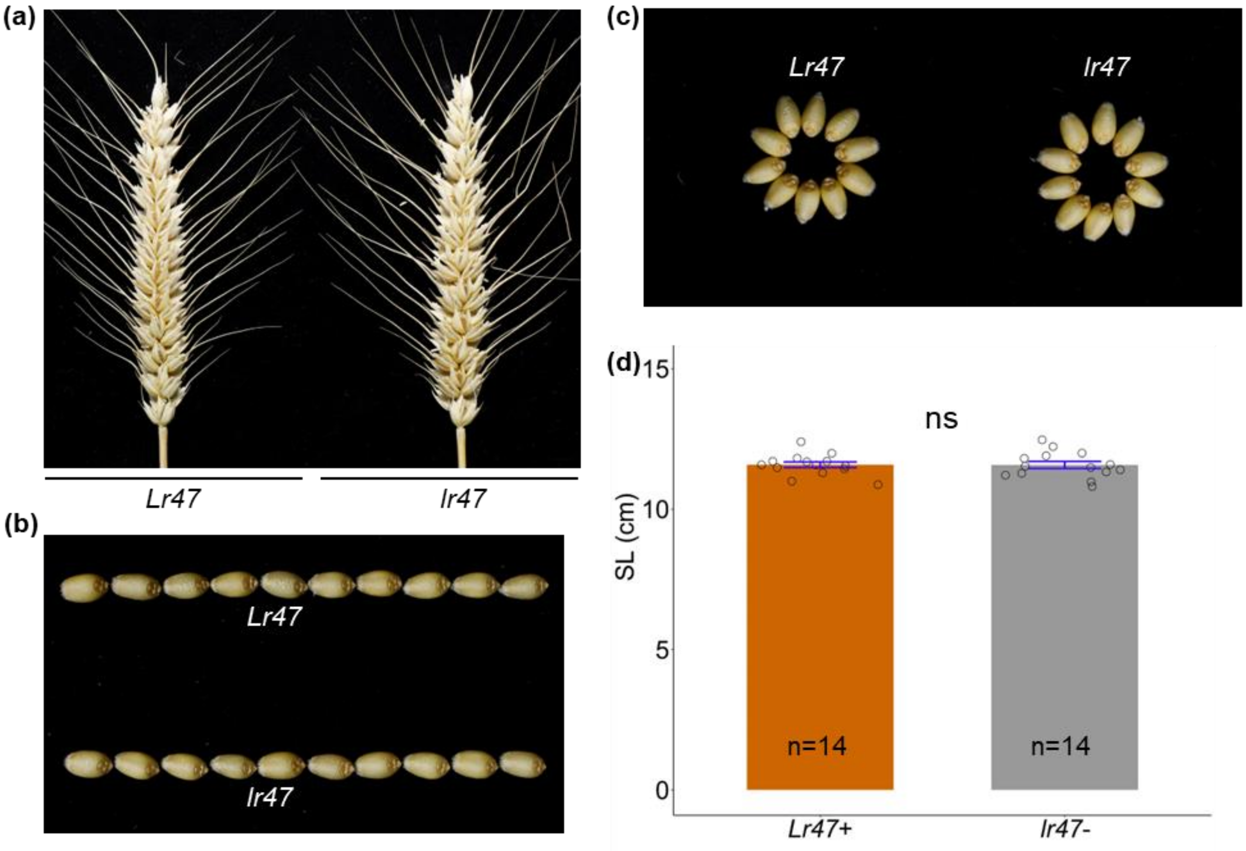

Supplementary Fig. 26. Statistical analysis of spike length between transgenic plants and the untransformed control Fielder under disease-free conditions. Representative spikes (**a**) and seeds (**b**, **c**) of *Lr47* transgenic plants and Fielder. (**d**) spike length (SL). Plants were grown in a controlled walk-in growth chamber at 24 °C day / 22 °C night with a 16 h light/8 h dark photoperiod. Homozygous T_2 transgenic plants were derived from one selected T_1 plant in T_1CS401-2 family. Gray open dots represent single data points. The significance of the differences was estimated using two-tailed *t*-test. Error bars are standard errors of the means. ns = not significant. Source data are provided as a Source Data file.

Supplementary Table 1. Avirulence/virulence formulae of *Pt* races used in this study. To determine the resistance profiles of *Lr47*, seedlings of *Lr47* NILs (Express *Lr47*, UC1041 *Lr47*, and RSI5 *Lr47*) and their recurrent parents (Express, UC1041, and RSI5) were challenged with these *Pt* races. *Lr47* is underlined.

Race	Origin	Avirulence	Virulence
FHJL	China	*Lr1 2a 2b 3ka 9 10 14a 18 19 20 21 24 28 29 30 36 42 47 51 53*	*Lr2c 3 3bg 11 13 14b 15 16 17 23 26 33 39 44 45 B*
PHQS	China	*Lr2a 2b 9 15 18 19 20 24 28 29 30 35 42 44 45 47 51 53*	*Lr1 2c 3 3bg 3ka 10 11 13 14a 14b 16 17 21 23 26 33 36 39 B*
FHJR	China	*Lr1 2a 2b 3ka 9 19 24 28 29 30 36 42 44 47 51 53*	*Lr2c 3 3bg 10 11 13 14b 15 16 17 18 20 21 23 26 33 39 45 B*
PHTT	China	*Lr2a 2b 9 15 19 20 24 28 29 44 47 51 53*	*Lr1 2c 3 3bg 3ka 4 10 11 13 14a 14b 16 17 18 21 23 26 30 33 35 36 39 42 45 B*
THTT	China	*Lr9 19 20 24 28 29 36 44 47 51 53*	*Lr1 2a 2b 2c 3 3bg 3ka 10 11 13 14a 14b 15 16 17 18 21 23 26 30 33 45 B*
FHJQ	China	*Lr1 2a 3ka 9 14a 15 18 19 20 21 24 28 29 30 36 42 47 51 53*	*Lr2b 2c 3 3bg 10 11 13 14b 16 17 23 26 33 39 44 45 B*
PHSS	China	*Lr9 15 19 20 24 18 28 29 30 47 53*	*Lr1 2b 2c 3 3bg 3ka 4 10 11 13 14a 14b 16 17 21 23 26 33 35 36 39 45 51*
FHSS	China	*Lr1 2a 9 18 19 24 29 30 36 39 47 51 53*	*Lr2b 2c 3 3bg 3ka 10 11 13 14a 14b 15 16 17 20 21 23 26 33 44 45 B*
PHTS	China	*Lr2a 2b 9 15 18 19 24 28 29 42 47 51 53*	*Lr1 2c 3 3bg 3ka 10 11 13 14a 14b 16 17 23 26 30 33 36 39 45 B*
KHSS	China	*Lr1 9 15 18 19 21 24 29 36 39 47 51 53*	*Lr2a 2b 2c 3 3bg 3ka 10 11 13 14b 16 17 23 26 30 33 44 45 B*
TGTS	China	*Lr9 14b 18 19 24 26 29 36 47 51 53*	*Lr1 2a 2b 2c 3 3bg 3ka 10 11 13 14a 15 16 17 20 21 23 30 33 39 44 45 B*
NHJS	China	*Lr2a 2b 3 3bg 3ka 9 15 18 19 21 24 28 29 30 42 47 51 53*	*Lr1 2c 10 11 13 14a 14b 16 17 23 26 33 36 39 44 45 B*
FRJS	China	*Lr1 2a 2b 3ka 15 18 19 24 28 29 30 39 47 51 53*	*Lr2c 3 3bg 10 11 13 14b 16 17 21 23 26 33 36 44 45 B*
FGBS	China	*Lr1 2a 3ka 9 11 15 17 18 19 21 24 26 28 29 30 39 47 51 53*	*Lr2b 2c 3 3bg 10 13 14b 16 23 33 36 44 45 B*
PGJS	China	*Lr2a 3ka 15 18 19 20 28 29 30 36 39 44 47 51 53*	*Lr1 2b 2c 3 3bg 10 11 13 14b 16 17 21 23 33 45 B*
THSP	China	*Lr9 10 19 21 24 28 29 30 36 44 47 51 53*	*Lr1 2a 2b 2c 3 3bg 3ka 11 13 14b 15 16 17 18 20 23 26 33 45 B*
SHJT	China	*Lr3 3ka 9 19 24 28 29 30 47 51 53*	*Lr1 2a 2b 2c 10 11 13 14a 14b 16 17 18 21 26 33 36 39 44 45 B*
PHJS	China	*Lr2a 2b 3ka 9 18 19 21 23 28 29 30 44 47 51 53*	*Lr1 2c 3 3bg 10 11 13 14a 14b 15 16 17 26 33 36 39 45 B*
FHGS	China	*Lr1 2a 3ka 9 17 18 19 29 30 42 47 51 53*	*Lr2b 2c 3 3bg 10 11 13 14a 14b 16 21 23 33 36 44 45 B*
FHGQ	China	*Lr1 2a 3 3a 3ka 9 14a 15 17 18 19 20 23 24 28 29 30 36 38 39 41 42 44 45 46 47 50 51 53*	*Lr2b 2c 3 3bg 4 10 11 13 14b 16 21 25 26 29 32 33 36 40 B*
THDB	China	*Lr3ka 9 10 11 14a 18 19 24 28 30 47 51 53 B*	*Lr1 2a 2c 3 14b 16 17 26 33 44*
PHRT	China	*Lr2a 9 17 19 24 28 47 51 53*	*Lr1 2c 3 3ka 10 11 14a 14b 16 18 26 30 33 44 B*
FHHM	China	*Lr1 2a 3ka 9 10 14a 17 19 24 28 47 51 53*	*Lr2c 3 11 14b 16 18 26 30 33 44 B*
HCJR	China	*Lr1 2c 3ka 9 14a 16 19 24 28 30 47 51 53*	*Lr2a 3 10 11 14b 17 18 26 33 44 B*

Supplementary Table 2. Summary of sequencing data. All the raw sequencing data and genome assembly generated for this project were deposited at the National Genomics Data Center under BioProject accession number CRA011051 that are publicly accessible at https://ngdc.cncb.ac.cn/gsa.

Wheat Lines	Total reads before filtering (million)	Reads after filtering (million)	Sequencing approaches	Function
Kern *Lr47*	2032.72	2003.918	whole genome re-sequencing	SNP calling and gene mapping
m118	1096.16	1084.49	whole genome re-sequencing	
Kern *Lr47*	259.138148	256.070506	RNA-seq	de novo assembly
m1649	87.795364	86.992922	RNA-seq	Mutant
m1541	69.86013	68.879952	RNA-seq	Mutant
m1576	80.37078	79.159946	RNA-seq	Mutant
m1599	70.66401	69.659082	RNA-seq	Mutant
m1606	82.000452	80.904274	RNA-seq	Mutant
m178	80.04645	79.306976	RNA-seq	Mutant
m118	91.238988	91.225154	RNA-seq	Mutant
m125	89.09825	89.080594	RNA-seq	Mutant
m152	82.013166	81.998536	RNA-seq	Mutant
m41	78.870744	78.855548	RNA-seq	Mutant
AE915	86.163098	85.557414	RNA-seq	SNP calling and source analysis
PI 542258	48.064094	47.664558	RNA-seq	
PI 542251	43.220682	42.878152	RNA-seq	
Y162	44.6323	44.282092	RNA-seq	
T2140002	49.575458	49.165758	RNA-seq	
PI 560751	46.846638	46.41047	RNA-seq	
PI 542260	50.231784	49.801002	RNA-seq	
PI 542270	46.547742	46.197444	RNA-seq	
AE1590	80.425106	79.845356	RNA-seq	
PI 554292	83.379176	82.931472	RNA-seq	

Supplementary Table 3. Estimated copy number of transgenic insertions. Number of insertions in each transgenic event was estimated based on the segregation ratio of T_1 plants and TaqMan copy number assays.

		T_1 Transgenic families										
		**T_1CS401-1**	**T_1CS401-2**	**T_1CS401-3**	**T_1CS401-4**	**T_1CS401-5**	**T_1CS401-6**	**T_1CS401-7**	**T_1CS401-8**	**T_1CS401-9**	**T_1CS401-10**	**T_1CS401-11**
Genotyping[1]	present *CNL2*	25	20	21	27	24	28	20	20	24	25	27
	absent *CNL2*	0	5	2	0	1	0	6	5	3	0	0
χ^2 *P* value	1 copy	0.0039	**0.5637**	0.0710	0.0027	0.0153	0.0023	**0.8208**	**0.5637**	0.0956	0.0039	0.0027
	2 copies	0.1967	0.0045	**0.6280**	0.1797	**0.6421**	0.1719	0.0004	0.0045	**0.2967**	0.1967	0.1797
	3 copies	**0.5287**	<0.0001	0.0058	0.5127	0.3258	0.5050	<0.0001	<0.0001	<0.0001	0.5287	0.5127
	4 copies	0.7542	<0.0001	<0.0001	**0.7449**	0.0038	**0.7404**	<0.0001	<0.0001	<0.0001	0.7542	**0.7449**
	5 copies	0.8758	<0.0001	<0.0001	0.8709	<0.0001	0.8686	<0.0001	<0.0001	<0.0001	**0.8758**	0.8709
TaqMan	Transgene[2] / Kern *Lr47*	3.19	1.23	1.92	3.96	2.43	3.94	0.93	1.33	1.91	5.24	4.27
Estimated copy No.		3	1	2	4	2	4	1	1	2	5	4

[1] Genotyped with the molecular marker *Lr47speF5R5* (Supplementary Data 3).

[2] Since T_0 transgenic plants were heterozygous, we divided the T_0s copy number values by 1/2*Kern *Lr47* copy number.

Supplementary Table 4. A collection of 24 accessions of *T. monococcum*, 78 of *T. turgidum*, 144 of *T. aestivum* (including six *Lr47* NILs), and 118 of *Ae. speltoides* was used to test the presence of *Lr47*. All these accessions were screened using the marker *Lr47mas*. The dominant marker *Lr47mas* could be a diagnostic marker for the presence of *Lr47* in *T. monococcum*, *T. turgidum* and *T. aestivum*. PI, CItr and GSTR numbers correspond to Germplasm Resources Information Network (GRIN) numbers.

Accessions	Accession No.	With / without *Lr47*
T. monococcum: PI 306545, PI 352505, PI 427498, CItr 2433, PI 272556, PI 427465, PI 352273, PI 427444, PI 427464, PI 573523, DV92, PI 306540, G3116, PI 168806, PI 272560, PI 511384, PI 245756, CItr 17674, PI 427507, PI 573520, PI 560720, PI 487249, PI 277121, PI 427580	24	without (-)
T. turgidum: CItr 7687, CItr 12213, CItr 12214, CItr 14133, CItr 14135, CItr 14621, CItr 14637, CItr 14916, CItr 14919, CItr 14970, PI 74108, PI 94615, PI 94616, PI 94624, PI 94626, PI 94631, PI 94635, PI 94638, PI 94656, PI 94657, PI 94664, PI 94666, PI 94674, PI 94675, PI 94747, PI 101971, PI 154582, PI 164578, PI 168673, PI 168679, PI 193877, PI 193879, PI 193880, PI 193882, PI 193883, PI 194042, PI 194375, PI 197259, PI 197260, PI 197481, PI 197485, PI 197486, PI 197489, PI 197492, PI 197493, PI 197495, PI 217637, PI 217639, PI 217640, PI 221400, PI 225332, PI 244341, PI 254147, PI 254164, PI 254165, PI 254189, PI 254190, PI 272531, PI 273978, PI 273980, PI 273981, PI 275996, PI 276013, PI 298582, PI 298586, PI 319869, PI 324076, PI 349043, PI 355477, PI 355507, PI 362696, PI 377655, PI 377656, PI 377657, PI 384332, PI 387683, PI 480460, PI 532305	78	without (-)
T. aestivum* (*Lr47* introgression lines): Yecora Rojo *Lr47*, PI 603918, Kern *Lr47*, UC1041 *Lr47*, Express *Lr47*, RSI5 *Lr47	6	with (+)
T. aestivum: PI 178759, PI 181434, PI 182527, PI 189747, PI 277012, PI 442904, PI 566596, PI 596533, PI 600683, PI 634936, PI 648417, PI 648419, PI 660056, PI 660057, PI 660059, PI 660060, PI 660064, PI 672538, PI 675564, PI 679598, PI 679603, PI 679605, PI 679621, Patwin, Clear white, Chinese Spring, Express, Fielder, Avocet S, Kern, RSI5, SY95-71, UC1041, UC1110, GSTR 409, GSTR 437, GSTR 441, Aikang58, Bainong207, Chuanmai32, Chuanmai42, Chuannong26, Chuannong30, Chuanyu25, Dan1412, Datang66, Gaoyou2018, Heng4399, Hongheshangtou, Huacheng3366, Huaimai22, Huaimai29, Huaimai33, Huaimai35, Huaimai40, Huaimai44, Jimai2, Jimai229, Jimai24, Jimai44, Jinan17, Liangken66, Liangken77, Longtang2, Luyuan502, Mianmai112, Mianmai1501, Mianmai285, Mianmai367, Mianmai37, Mianmai51, Mianmai902, Ningmai13, Nongda3753, Nongda399, Nongda5321, Qingnong2, Ruihua520, Shannong20, Shannong24, Shannong28, Shannong29, Shannong45, Shiluan02-1, Shinong086, Shixin828, Shumai133, Shumai830, Shumai969, Sumai3, Taichang29, Tainong18, Taishan28, Taimai198, Wanfeng269, Wangshuibai, Xiaoyan22, Xiaoyan9, Xikemai10, Xikemai8, Xikemai9, Xinmai26, Xinong979, Xumai9158, Yangfumai4, Yangmai158, Yangmai16, Yangmai21, Yangmai23, Yannong19, Yannong999, Yingbo700, Yuemai6, Yumai70, Zhengmai0856, Zhengmai0926, Zhengmai101, Zhengmai1342, Zhengmai1354, Zhengmai136, Zhengmai1860, Zhengmai369, Zhengmai379, Zhengmai618, Zhengmai6687, Zhengmai6694, Zhengmai7698, Zhengmai9023, Zhengmai925, Zhengzhou5389, Zhenmai10, Zhenmai168, Zhongkemai142, Zhongmai578, Zhongmai895, Zhoumai18, Zhoumai22, Zhoumai27	138	without (-)
***Ae. speltoides*: T2140002, Y162, Y397**	3	with (+)
Ae. speltoides: T2140004, T2140006, AE915, AE1590, PI 369663, PI 554292, PI 486264, PI 542238, PI 554304, IG48766, IG48996, PI 542270, PI 542260, PI 542264, PI 560529, PI 560751, PI 560528, PI 542252, IG48765, IG48847, T2140003, PI 369584, PI 369585, PI 369586, PI 369587, PI 369588, PI 369589, PI 369590, PI 369591, PI 369592, PI 369593, PI 369594, PI 369595, PI 369596, PI 369597, PI 369598, PI 369604, PI 369605, PI 369606, PI 369607, PI 369608, PI 369609, PI 369610, PI 369611, PI 369615, PI 369618, PI 369621, PI 369624, PI 369665, PI 449341, PI 487231, PI 487233, PI 554295, PI 573452, PI 542241, PI 542243, PI 542245, PI 542251, PI 542258, PI 560527, PI 542268, PI 542274, PI 560748, Ae48, Ae50, T2140008, T2140005, PI 369622, PI 573449, PI 573450, PI 573452, PI 554303, PI 560752, PI 542254, PI 449338, PI 254865, PI 170203, PI 560747, PI 542239, PI 542242, PI 542244, PI 542246, PI 542259, PI 542261, PI 542262, PI 542265, PI 542266, PI 542267, PI 542271, PI 542272, PI 542276, PI 560530, PI 487232, PI 487234, PI 486263, PI 393495, PI 266817, PI 554296, PI 554297, PI 554300, PI 560749, PI 542247, PI 542248, PI 542249, PI 542253, PI 542257, PI 542263, PI 449339, PI 449340, PI 393492, PI 393493, PI 174010, PI 173614, PI 172685, PI 487238	115	without (-)

Supplementary Table 5. Near isogenic *Lr47* lines (NILs) and their recurrent cultivars used in this study. PI and CItr numbers correspond to Germplasm Resources Information Network (GRIN) numbers.

Line	Accession	Pedigree	Resistance gene *Lr47*	References
Pavon	PI 519847	Vicam//Ciano sib/Siete Cerros 66/3/Kalyansona/Bluebird	-	3
Pavon *Lr47*	PI 603918	Pavon*8//T7AS-7S#1S-7S#1S/ph1b	+	3
Kern	PI 612142	Tadorna/Inia 66//Yecora Rojo/3/Klasic	-	4
Kern *Lr47*	PI 638739	PI603918*7/Kern	+	4
Yecora Rojo	CItr 17414	Ciano 67//Sonora 64/Klein Rendidor/3/II-8156 = II23584	-	5
Yecora Rojo *Lr47*	PI 638738	PI603918*7/Yecora Rojo	+	4
Express	PI 573003	Veery/BH1146	-	4
Express *Lr47*	-	PI603918*7/Express	+	4
RSI5	-	Tadinia/Probrand 775//23IBWSN#76	-	4
RSI5 *Lr47*	-	PI603918*7/RSI5	+	4
UC1041	-	Yecora Rojo/Tadinia	-	4
UC1041 *Lr47*	-	PI603918*7/UC1041	+	4

Supplementary references

1 Robinson, J. T. *et al.* Integrative genomics viewer. *Nat Biotechnol* **29**, 24-26 (2011).

2 Jumper, J. *et al.* Highly accurate protein structure prediction with AlphaFold. *Nature* **596**, 583-589 (2021).

3 Dubcovsky, J., Lukaszewski, A., Echaide, M., Antonelli, E. & Porter, D. Molecular characterization of two *Triticum speltoides* interstitial translocations carrying leaf rust and greenbug resistance genes. *Crop Sci* **38**, 1655-1660 (1998).

4 Brevis, J. C. *et al.* Agronomic and quality evaluation of common wheat near-isogenic lines carrying the leaf rust resistance gene *Lr47*. *Crop Sci* **48**, 1441-1451 (2008).

5 Qualset, C.O., Synthesis, breeding, adaptation, and utilization of *triticale* in California. *Triticale* **9**, 71-82 (1985).
