# Supplementary material for: Cloning of the wheat leaf rust resistance gene Lr47 introgressed from Aegilops speltoides
Source: Nat Commun. 2023 Sep 28;14:6072. doi: 10.1038/s41467-023-41833-2 (PMC10539295; doi:10.1038/s41467-023-41833-2)
Supplement: Supplementary file 3 — Description of Additional Supplementary Files [file 41467_2023_41833_MOESM3_ESM.pdf]

Description of Additional Supplementary Files

File Name: Supplementary Data 1
Description: Geographic distribution of *Ae. speltoides* accessions and their reactions against *Pt* race PHQS.

File Name: Supplementary Data 2
Description: Putative *Ae. speltoides*-specific SNPs.

File Name: Supplementary Data 3
Description: Primer sequences used in the study.

File Name: Supplementary Data 4
Description: Predicted genes within the candidate region based on the genomic sequence of Chinese Spring RefSeqv1.1.

File Name: Supplementary Data 5
Description: Predicted genes within the candidate region based on the reference genome of the *Ae. speltoides* ‘TS01’.

File Name: Supplementary Data 6
Description: 199 polymorphic sites used to generate the Neighbor-Joining tree.
