# Supplementary material for: Cloning of the wheat leaf rust resistance gene Lr47 introgressed from Aegilops speltoides
Source: Nat Commun. 2023 Sep 28;14:6072. doi: 10.1038/s41467-023-41833-2 (PMC10539295; doi:10.1038/s41467-023-41833-2)
Supplement: Supplementary file 10 — Reporting Summary [file 41467_2023_41833_MOESM10_ESM.pdf]

Corresponding author(s): Shisheng Chen
Xiaodong Wang

Last updated by author(s): Sep 17, 2023

Reporting Summary

Nature Portfolio wishes to improve the reproducibility of the work that we publish. This form provides structure for consistency and transparency in reporting. For further information on Nature Portfolio policies, see our Editorial Policies and the Editorial Policy Checklist.

Statistics

For all statistical analyses, confirm that the following items are present in the figure legend, table legend, main text, or Methods section.

n/a	Confirmed	
☐	☒	The exact sample size (*n*) for each experimental group/condition, given as a discrete number and unit of measurement
☐	☒	A statement on whether measurements were taken from distinct samples or whether the same sample was measured repeatedly
☐	☒	The statistical test(s) used AND whether they are one- or two-sided<br>*Only common tests should be described solely by name; describe more complex techniques in the Methods section.*
☒	☐	A description of all covariates tested
☒	☐	A description of any assumptions or corrections, such as tests of normality and adjustment for multiple comparisons
☐	☒	A full description of the statistical parameters including central tendency (e.g. means) or other basic estimates (e.g. regression coefficient) AND variation (e.g. standard deviation) or associated estimates of uncertainty (e.g. confidence intervals)
☐	☒	For null hypothesis testing, the test statistic (e.g. *F*, *t*, *r*) with confidence intervals, effect sizes, degrees of freedom and *P* value noted<br>*Give P values as exact values whenever suitable.*
☒	☐	For Bayesian analysis, information on the choice of priors and Markov chain Monte Carlo settings
☒	☐	For hierarchical and complex designs, identification of the appropriate level for tests and full reporting of outcomes
☒	☐	Estimates of effect sizes (e.g. Cohen's *d*, Pearson's *r*), indicating how they were calculated

Our web collection on statistics for biologists contains articles on many of the points above.

Software and code

Policy information about availability of computer code

Data collection: No software was used to collect the phenotype data

Data analysis:

Here are software packages used in the study.
RNA-seq data analysis, MutRNASeq, and mapping: Trimmomatic version 0.32; STAR v2.7.10a; IGV version 2.8.9; Freebayes v1.3.6; BCFtools v1.14; Spades version 3.14.1; SAMtools v1.8; BWA110 v0.7.12; Integrative Genomics Viewer (IGV) version 2.8.9;

Genome-specific primers: Primer 3 v0.4.0;

Phylogenetic analysis: MEGA v7.0; iTOL v5.0 (https://itol.embl.de/); NCBI (ttps://www.ncbi.nlm.nih.gov); Reference genomes (https://wheat.pw.usda.gov/blast/);

Structure prediction: AlphaFold v2.0.1;

Evaluation of agronomic and quality traits: Wanshen SC-G; Instron 5848 Microtester; Model SpectraStar 2600 XT-R; TA-XT-plus Texture Analyzer;

Other softwares used for data analysis: ESPript 3.0 (https://espript.ibcp.fr/ESPript/cgi-bin/ESPript.cgi); cNLS Mapper (https://nls-mapper.iab.keio.ac.jp/cgi-bin/NLS_Mapper_form.cgi); Pfam (http://pfam-legacy.xfam.org/search/sequence).

For manuscripts utilizing custom algorithms or software that are central to the research but not yet described in published literature, software must be made available to editors and reviewers. We strongly encourage code deposition in a community repository (e.g. GitHub). See the Nature Portfolio guidelines for submitting code & software for further information.

Data

Policy information about availability of data

All manuscripts must include a data availability statement. This statement should provide the following information, where applicable:

- Accession codes, unique identifiers, or web links for publicly available datasets
- A description of any restrictions on data availability
- For clinical datasets or third party data, please ensure that the statement adheres to our policy

Data supporting the findings of this work are available within the paper and its supplementary information files. All the raw sequencing data for this project are archived at the National Genomics Data Center under BioProject accession number PRJCA016987. The transcriptome assembly was deposited in Figshare (https://doi.org/10.6084/m9.figshare.23937879). The sequence of the Lr47 gene was deposited in NCBI Genbank under accession number OQ919262. Source data are provided with this paper.
The following public databases/datasets were used in the study: Chinese Spring reference genome (IWGSC, 2018); the other published reference genomes of wheat and its wild relatives https://wheat.pw.usda.gov/blast/ and http://202.194.139.32/.

Research involving human participants, their data, or biological material

Policy information about studies with human participants or human data. See also policy information about sex, gender (identity/presentation), and sexual orientation and race, ethnicity and racism.

Reporting on sex and gender	N/A
Reporting on race, ethnicity, or other socially relevant groupings	N/A
Population characteristics	N/A
Recruitment	N/A
Ethics oversight	N/A

Note that full information on the approval of the study protocol must also be provided in the manuscript.

Field-specific reporting

Please select the one below that is the best fit for your research. If you are not sure, read the appropriate sections before making your selection.

☒ Life sciences ☐ Behavioural & social sciences ☐ Ecological, evolutionary & environmental sciences

For a reference copy of the document with all sections, see nature.com/documents/nr-reporting-summary-flat.pdf

Life sciences study design

All studies must disclose on these points even when the disclosure is negative.

Sample size	No sample-size calculation was chosen. For high-resolution genetic mapping, we used 2,654 plants and another 1,141 plants from the second population (a total of 7,590 gametes). This number represents what we could practically handle while striving to reduce the mapping interval as much as possible. The population size is bigger than most reported population sizes in similar studies (Lin et al. 2022, Nat. Commun., 13:3044; Luo et al. 2022, Plant Biotechnol J, 20: 554-563).
Data exclusions	No data were excluded.
Replication	All experiments were repeated at least twice and multiple biological replicates were used in all experiments.
Randomization	We randomized mutant lines and wild type controls when phenotyping as is normal practice. When phenotyping transgenic plants, 25 seeds were randomly chosen from each T1 family. It is not relevant to other experiments as no subjects were allocated to experimental groups.
Blinding	Blinding was performed when phenotyping the mutant lines / transgenic plants, qRT-PCR for determining transgene expression, and cytogenetic assays. It is not performed when carrying out phylogenetic analysis, sequencing analysis, protein structure analysis, and function analysis as required by the experiments.

Reporting for specific materials, systems and methods

We require information from authors about some types of materials, experimental systems and methods used in many studies. Here, indicate whether each material, system or method listed is relevant to your study. If you are not sure if a list item applies to your research, read the appropriate section before selecting a response.

Materials & experimental systems

n/a	Involved in the study	
☐	☒	Antibodies
☒	☐	Eukaryotic cell lines
☒	☐	Palaeontology and archaeology
☒	☐	Animals and other organisms
☒	☐	Clinical data
☒	☐	Dual use research of concern
☒	☐	Plants

Methods

n/a	Involved in the study	
☒	☐	ChIP-seq
☒	☐	Flow cytometry
☒	☐	MRI-based neuroimaging

Antibodies

Antibodies used	Commercial antibodies: Anti-GFP (Abcam, Cambridge, UK; Catalog No. ab290; Lot No. GR3431263-1; 1:2500 dilution); Goat Anti-Rabbit IgG-HRP (Abmart, Shanghai, China; Catalog No. M21002S; Lot No.334666; 1:8000 dilution).
Validation	Validation of commercial antibodies is provided on the manufacturer's website: Anti-GFP (https://www.abcam.com/products/primary-antibodies/gfp-antibody-ab290.html); Goat Anti-Rabbit IgG-HRP (http://www.ab-mart.com.cn/page.aspx?node=%2062%20&id=%20980).
